# Prevalence and Age at Diagnosis of Risk Factors for Severe Respiratory Syncytial Virus Disease Among US Adults: An Analysis of 2011–2020 NHANES Data

**DOI:** 10.1111/irv.70181

**Published:** 2025-11-30

**Authors:** Emily K. Horn, David Singer, Alison Booth, Hai Nguyen, Cynthia Saiontz‐Martinez, Ariel Berger

**Affiliations:** ^1^ GSK Philadelphia Pennsylvania USA; ^2^ Evidera PPD Basel Switzerland; ^3^ Evidera PPD London UK; ^4^ Evidera PPD Bethesda Maryland USA; ^5^ Evidera PPD Waltham Massachusetts USA

**Keywords:** age of onset, health disparities, National Health and Nutrition Examination Survey, primary prevention, respiratory syncytial virus, social determinants of health, United States, vaccination

## Abstract

**Background:**

Respiratory syncytial virus (RSV) vaccines are approved in the United States (US) among adults ≥ 60 years of age (YoA). Understanding disparities in risk factors (RFs) for severe RSV disease by social determinants of health is crucial for equitable RSV vaccination among at‐risk adults. We investigated diagnosed/undiagnosed RF prevalence, age at RF diagnosis/identification, and association of key characteristics with RF diagnosis/identification among US adults.

**Methods:**

We analyzed 2011–2020 National Health and Nutrition Examination Survey (NHANES) data to calculate RF prevalence and mean age at RF diagnosis/identification. Multivariable regression models were developed to estimate associations between characteristics and RF diagnosis/identification. Results were weighted to reflect the US noninstitutionalized adult population ≥ 20 YoA.

**Results:**

After weighting, the analysis included a sample of 233,118,491 adults. Overall, 28.0% had ≥ 1 diagnosed RF (12.8% pulmonary; 9.2% cardiovascular; 14.1% endocrine/metabolic). Undiagnosed diabetes and renal disease were identified in 3.7% and 12.5% of adults, respectively. Among those with ≥ 1 diagnosed RF, mean age at RF diagnosis was 40.1 years and 60.0% were diagnosed before 50 YoA. Increasing age, belonging to a racial or ethnic minority group, and lower income were significantly associated with having ≥ 1 RF.

**Conclusions:**

Approximately one in four adults had ≥ 1 diagnosed RF for RSV, with most diagnosed before 50 YoA. Adults in racial and ethnic minority and socioeconomically disadvantaged groups were more likely to have RFs and be diagnosed at younger ages. Efforts are needed to ensure all US adults at increased risk for severe RSV disease have access to RSV vaccination.

## 1 Introduction

Respiratory syncytial virus (RSV) is a common seasonal respiratory virus that infects the lungs and airways [1]. Among adults, RSV commonly results in upper respiratory tract disease (URTD) that resolves within 1–2 weeks [1]. However, RSV infection can result in lower respiratory tract disease (LRTD), causing substantial disease burden or even death, with risk of more severe RSV disease being higher among older adults and adults with certain underlying medical conditions [1].

In the United States (US), a landmark prospective cohort study found that 3%–7% of healthy adults aged ≥ 65 years and 4%–10% of high‐risk adults (aged ≥ 21 years with cardiopulmonary disease) develop RSV infection annually [2]. The impact of these infections is substantial, including more than 177,000 estimated hospitalizations and 14,000 estimated deaths among adults aged ≥ 65 years in the US each year [2]. Additionally, a multi‐hospital‐system study conducted in New York from 2017 to 2020 estimated that rates of RSV‐related hospitalization were 4–13 and 4–6 times higher among adults aged ≥ 65 years with chronic obstructive pulmonary disease (COPD) and coronary artery disease (CAD), respectively, and 4–8 times higher among adults aged ≥ 60 years with congestive heart failure (CHF), than those without these conditions [3].

As of 2025, vaccines against RSV are available and approved by the Food and Drug Administration (FDA) for all adults aged ≥ 60 years and for younger adults aged < 60 years who are at increased risk of RSV‐related LRTD (AREXVY, GSK; ABRYSVO, Pfizer Inc.; MRESVIA, Moderna Inc.) [4, 5, 6]. The US Centers for Disease Control and Prevention's (CDC's) Advisory Committee on Immunization Practices (ACIP) recommends RSV vaccination for all adults aged ≥ 75 years, and adults aged 50–74 years who are at increased risk of RSV‐LRTD, as of June 2025 [7]. However, this risk‐based recommendation does not include younger at‐risk adults aged < 50 years, and it limits access to RSV vaccination among those aged 50–74 years who remain undiagnosed with medical conditions associated with increased risk of severe RSV disease. Uptake of RSV vaccines is notably low even among eligible individuals aged ≥ 60 years in the US (15.3% among those aged 60–74 years at increased risk and 21.3% among all adults aged ≥ 75 years, as per a recent analysis of claims data from August 2023 to February 2025) [8], rendering a large proportion of individuals unprotected against RSV infection and severe RSV disease.

Racial and ethnic minority populations and populations experiencing socioeconomic disadvantage may more commonly have chronic medical conditions [9, 10]. Moreover, existing data suggest that chronic medical conditions may be more likely to remain undiagnosed among racial and ethnic minority groups due to disparities in healthcare access [11, 12]. Therefore, the current RSV vaccination recommendations may not enable access to RSV vaccination for adults who are disproportionally at increased risk of severe RSV disease.

To support equitable RSV vaccination among at‐risk adults, it is important to first understand the degree to which disparities exist in the prevalence of underlying medical conditions associated with increased risk for severe RSV disease, i.e., risk factors (RFs), within various racial and ethnic minority and socioeconomically disadvantaged groups. Currently, there are no nationally representative data that account for the co‐prevalence of these RFs among adults, and there are sparse data on the age at RF diagnosis stratified by social determinants of health (SDOH). Previous research using National Health and Nutrition Examination Survey (NHANES) data found that age at diagnosis of hypertension, cardiovascular disease (CVD), and diabetes was 4–5 years, 6–8 years, and 5–7 years earlier in non‐Hispanic Black and Hispanic adults than in non‐Hispanic White adults, respectively [13, 14, 15]; however, other medical conditions have not been evaluated, and data are needed on the proportion of adults diagnosed with RFs by age thresholds of current RSV vaccination recommendations.

This study used data from several cycles of NHANES to examine the prevalence and age at diagnosis of individual and collective known RFs for severe RSV disease in the US, and the association between certain SDOH and the presence and age at diagnosis of ≥ 1 RF. Findings may help to inform policymakers, payers, and other population‐based decision‐makers in their efforts to reduce disparities in RSV vaccination and RSV‐related disease burden.

## 2 Methods

### 2.1 Study Design and Data Source

This retrospective study was a cross‐sectional analysis of NHANES data, collected by the National Center for Health Statistics (NCHS) of the CDC, which examines the demographic, health, and nutritional status of US noninstitutionalized adults (aged ≥ 20 years) [16]. Each NHANES cycle collects data from three modules: personal interviews, standardized physical examination, and laboratory specimen collection.

In this study, outcomes of interest included 10 chronic medical conditions known to be associated with increased risk of severe RSV disease (referred to as RFs throughout this manuscript) [3, 17, 18], collected by NHANES over the cycles of interest, including pulmonary conditions (COPD [including emphysema and chronic bronchitis] and asthma), cardiovascular conditions (CHF, coronary heart disease [CHD], stroke, angina pectoris, and myocardial infarction [MI]), and endocrine and metabolic conditions (diabetes, renal disease, and liver disease). Of these 10 conditions, NHANES survey questions ascertained the current (as of the date of study assessment) presence of asthma, chronic bronchitis, and liver disease via follow‐up questions confirming whether respondents still have the condition at the point of participation in the survey.

Diagnosed RFs were described based on self‐report in the interview component of NHANES. Where applicable laboratory and/or examination measures were available (limited to diabetes and renal disease), undiagnosed RFs were based on positive examination/laboratory findings indicative of the condition in the absence of corresponding self‐reported diagnosis in NHANES. Diabetes was defined as plasma fasting glucose > 126 mg/dL (≥ 7.0 mmol/L), or hemoglobin A1C (HbA1C) > 6.5%, or a 2‐h oral glucose tolerance test result ≥ 200 mg/dL [19]. Renal disease was defined as estimated glomerular filtration rate (eGFR) < 60 mg/dL (Chronic Kidney Disease Epidemiology Collaboration [CKD‐EPI] formula) or urine albumin creatinine ratio ≥ 30 mg/g [20]. Overall results (including diagnosed and undiagnosed RFs) were identified using both questionnaire responses and findings from the examination/laboratory component. Age at diagnosis for diagnosed RFs reflected self‐reported age at diagnosis in the interview component; age at identification of undiagnosed RFs reflected age at NHANES screening. A summary of RFs for severe RSV disease that were capturable based on information obtained via the NHANES questionnaire and laboratory components, as well as NHANES questions related to age at diagnosis, is available in Appendix A.

### 2.2 Study Population

The source population included all individuals aged ≥ 20 years from the four most recent NHANES cycles, as of 2024: calendar year (CY) 2011–CY2012, CY2013–CY2014, CY2015–CY2016, and CY2017–March 2020. Due to the coronavirus disease 2019 (COVID‐19) pandemic, the CY2019–CY2020 cycle was not completed; instead, data from CY2019–March 2020 were combined with the CY2017–CY2018 cycle. Across applicable cycles, 26,280 individuals aged ≥ 20 years completed the interview, 24,925 (94.8%) completed the examination, and 23,726 (90.3%) completed the laboratory (and hence all three) components.

NHANES conducts in‐person health interviews and performs health measurements in mobile examination centers, which travel to communities throughout the US; using a list of households in selected neighborhoods, NHANES randomly chooses a small number for the survey, contacts every selected household, and utilizes a computer program to determine who in the household is eligible to participate [21]. To obtain reliable statistics, NHANES over‐samples adults aged ≥ 60 years and non‐Hispanic Black, non‐Hispanic Asian, and Hispanic individuals, and creates weights that project survey participants to total US noninstitutionalized adult (aged ≥ 20 years) population counts from the US Census Bureau [16]. Weights were applied in accordance with NHANES published guidelines to incorporate survey design complexities and allow extrapolation of findings [22, 23].

### 2.3 Data Analysis

To assess co‐prevalence of RFs, pooled, weighted analyses were conducted using SAS 9.4 (SAS, Cary, NC) to estimate the prevalence of ≥ 1 diagnosed RF, ≥ 1 undiagnosed RF, and ≥ 1 overall RF (diagnosed or undiagnosed). Additionally, among the subset of individuals with ≥ 1 RF, analyses estimated the mean age at diagnosis or identification of RFs and the percentage of adults diagnosed with RFs before age 50 and 60 years. Results are presented overall and by subgroups stratified by select self‐reported demographic variables: age, race and ethnicity (i.e., non‐Hispanic White, Hispanic, non‐Hispanic Black, and non‐Hispanic Asian), and income (measured as poverty income ratio [PIR]). NHANES estimates PIR by dividing family or individual income by the US Health and Human Services (HHS) poverty guidelines specific to the survey year. Values range from 0, representing no income, to ≥ 5, representing individual/family income of 5 or more times the federal poverty level; a ratio of < 1 represents individual/family income below federal poverty level [24].

Prevalence estimates were provided with their corresponding two‐sided 95% confidence intervals (CIs), which were calculated using the Clopper–Pearson method adapted for complex surveys [25, 26]. Pairwise tests were used to examine between‐subgroup differences in the prevalence of RFs and diagnosis of RFs before age 50 years (Rao–Scott–Chi–square tests) and in mean age at diagnosis of RFs between subgroups (Wald tests), respectively.

Univariable and multivariable logistic regression models were developed to estimate associations between selected characteristics and prevalence of ≥ 1 RF, as well as the diagnosis of ≥ 1 RF before age 50 and 60 years. Covariates for potential model inclusion consisted of age group at survey participation, gender, race and ethnicity, PIR, highest level of education, health insurance status, most frequented place for healthcare, employment status, smoking status, and body mass index. A correlation matrix was used to determine if intercorrelation existed between potential covariates of interest, leading to highest level of education attained and employment status being removed from both models and health insurance status being removed from the age at diagnosis model. Retained covariates with *p*‐values ≤ 0.10 in univariable analyses based on the corresponding Wald test were considered for inclusion in the multivariable models, in addition to covariates considered clinically relevant (i.e., strong association with the outcome based on existing literature).

### 2.4 Ethics

This study complied with all applicable laws regarding subject privacy. No direct subject contact or primary collection of individual human subject data occurred. The use of NHANES data does not require either informed consent or institutional review board approval.

## 3 Results

### 3.1 Participant Characteristics

After weighting the CY2011–March 2020 NHANES survey respondents to the US population, the analysis included a projected total sample of 233,118,491 adults aged ≥ 20 years. Almost one‐half of the sample (46.3%) were aged ≥ 50 years, 51.9% identified as female, and 64.3% as non‐Hispanic White. Almost one‐quarter of adults reported a PIR ≥ 5 (24.3%), while 13.5% reported a PIR of < 1 (Table S1).

### 3.2 Prevalence of Diagnosed RFs for Severe RSV Disease

The proportion of adults with ≥ 1 diagnosed RF identified through the interview component is reported overall and stratified by race and ethnicity and by PIR in Table 1. Overall, 28.0% (95% CI: 27.0%–29.1%) of adults had ≥ 1 diagnosed RF, with 12.8% (12.1%–13.5%) having ≥ 1 pulmonary RF, 9.2% (8.6%–9.8%) having ≥ 1 cardiovascular RF, and 14.1% (13.5%–14.7%) having ≥ 1 endocrine/metabolic RF. The prevalence of diagnosed RFs increased with age, across all racial and ethnic and PIR subgroups (Table 2). A total of 31.4% of adults aged 50–59 years and 46.9% of adults aged ≥ 60 years had ≥ 1 diagnosed RF for severe RSV disease.

**TABLE 1:** Percentage of adults aged ≥ 20 years with diagnosed RFs for severe RSV disease.

RFs, % (95% CI)	Overall and by race and ethnicity				
	Overall (*N* = 233,118,491)	Non‐Hispanic White (*N* = 150,001,228)	Hispanic (*N* = 35,427,374)	Non‐Hispanic Black (*N* = 26,693,818)	Non‐Hispanic Asian (*N* = 13,178,978)
**≥ 1 RF** ^**a**^	**28.0 (27.0–29.1)**	**29.0 (27.5–30.4)**	**23.3** * **(21.9–24.8)**	**30.3 (28.7–31.8)**	**19.6** * **(17.9–21.3)**
1 RF	18.1 (17.3–18.9)	18.3 (17.3–19.3)	16.9 (15.7–18.1)	19.7 (18.4–20.9)	14.3* (12.7–16.0)
2 RFs	5.7 (5.3–6.1)	5.9 (5.3–6.5)	4.0* (3.5–4.6)	6.4 (5.7–7.0)	3.4* (2.7–4.0)
3 RFs	2.2 (2.0–2.4)	2.4 (2.1–2.7)	1.3* (1.0–1.6)	2.7 (2.3–3.0)	1.1* (0.7–1.5)
≥ 4 RFs	2.0 (1.8–2.3)	2.4 (2.0–2.7)	1.1* (0.9–1.4)	1.6* (1.3–1.9)	0.7* (0.5–1.0)
**Pulmonary and cardiovascular**	**19.3 (18.5–20.2)**	**21.2 (19.9–22.4)**	**12.5** * **(11.5–13.5)**	**20.5 (19.3–21.7)**	**9.3** * **(8.1–10.5)**
**Pulmonary**	**12.8 (12.1–13.5)**	**14.0 (13.0–15.0)**	**8.2** * **(7.4–9.1)**	**13.6 (12.6–14.6)**	**5.3** * **(4.4–6.3)**
COPD^b^	6.0 (5.5–6.6)	7.2 (6.3–8.0)	2.4* (2.0–2.9)	5.0* (4.2–5.8)	1.6* (1.2–2.0)
Asthma (current)^c^	8.8 (8.3–9.4)	9.2 (8.3–10.0)	6.8* (6.1–7.6)	10.5* (9.6–11.4)	4.7* (3.8–5.5)
**Cardiovascular**	**9.2 (8.6–9.8)**	**10.2 (9.3–11.0)**	**5.4** * **(4.7–6.0)**	**9.5 (8.7–10.3)**	**4.6** * **(3.8–5.5)**
CHF	2.6 (2.3–2.9)	2.8 (2.4–3.1)	1.6* (1.2–1.9)	3.5* (3.0–4.1)	1.0* (0.7–1.3)
CHD	3.7 (3.2–4.2)	4.5 (3.8–5.3)	1.8* (1.4–2.2)	2.1* (1.8–2.4)	2.0* (1.5–2.5)
Stroke	3.2 (2.9–3.4)	3.3 (2.9–3.7)	1.9* (1.6–2.3)	4.2* (3.7–4.7)	1.5* (1.0–1.9)
Angina pectoris	2.2 (2.0–2.4)	2.7 (2.3–3.0)	1.2* (0.9–1.4)	1.4* (1.1–1.7)	1.1* (0.7–1.6)
MI	3.5 (3.1–3.8)	4.0 (3.4–4.5)	1.9* (1.5–2.3)	2.9* (2.5–3.2)	1.4* (1.0–1.8)
**Endocrine and metabolic**	**14.1 (13.5–14.7)**	**13.5 (12.8–14.3)**	**14.4 (13.3–15.6)**	**16.0** * **(14.9–17.1)**	**13.3 (12.1–14.5)**
Diabetes^d^	10.6 (10.1–11.1)	9.8 (9.1–10.5)	11.2* (10.1–12.2)	13.4* (12.4–14.3)	10.4 (9.3–11.6)
Renal disease	3.0 (2.7–3.3)	3.0 (2.6–3.5)	2.7 (2.3–3.1)	3.7 (3.3–4.2)	1.9* (1.3–2.5)
Liver disease (current)^e^	2.2 (2.0–2.5)	2.3 (2.0–2.7)	2.4 (2.0–2.8)	1.1* (0.8–1.3)	2.3 (1.8–2.7)

RFs, % (95% CI)	By PIR					
	≥ 5 (*N* = 56,705,042)	4–< 5 (*N* = 21,649,397)	3–< 4 (*N* = 26,718,652)	2–< 3 (*N* = 32,092,050)	1–< 2 (*N* = 43,133,903)	< 1 (*N* = 31,377,565)
**≥ 1 RF** ^**a**^	**21.0 (19.0–23.0)**	**23.7 (21.1–26.4)**	**26.2** * **(23.3–29.2)**	**30.2** * **(27.8–32.5)**	**33.4** * **(31.4–35.3)**	**33.8** * **(31.5–36.2)**
1 RF	15.1 (13.5–16.7)	15.5 (13.3–17.8)	17.9* (15.5–20.3)	19.2* (17.4–21.0)	20.0* (18.8–21.3)	20.4* (19.0–21.8)
2 RFs	3.9 (3.1–4.7)	4.9 (3.3–6.5)	5.1 (4.0–6.2)	6.1* (4.9–7.3)	7.1* (6.1–8.0)	7.2* (6.3–8.1)
3 RFs	1.0 (0.7–1.4)	1.7 (0.9–2.5)	1.8* (1.2–2.4)	2.3* (1.6–3.0)	3.1* (2.6–3.6)	3.0* (2.4–3.7)
≥ 4 RFs	0.9 (0.4–1.4)	1.6 (0.8–2.4)	1.5 (1.0–2.0)	2.5* (1.6–3.4)	3.2* (2.5–3.8)	3.2* (2.4–3.9)
**Pulmonary and cardiovascular**	**13.7 (11.7–15.7)**	**16.2 (13.8– 18.5)**	**17.4** * **(14.9– 20.0)**	**21.3** * **(19.5– 23.1)**	**24.0** * **(22.2– 25.9)**	**24.2** * **(22.2– 26.1)**
**Pulmonary**	**8.8 (7.1–10.4)**	**11.2 (9.2–13.1)**	**11.6** * **(9.6–13.7)**	**13.3** * **(11.8–14.8)**	**15.7** * **(14.2–17.3)**	**17.6** * **(15.9–19.3)**
COPD^b^	2.8 (2.1–3.6)	4.0 (2.9–5.2)	5.2* (4.0–6.5)	6.2* (4.8–7.6)	9.0* (7.7–10.4)	9.3* (7.7–10.8)
Asthma (current)^c^	6.8 (5.2–8.4)	8.6 (6.9–10.3)	7.7 (6.1–9.3)	9.1* (7.8–10.4)	9.8* (8.6–10.9)	12.2* (11.0–13.5)
**Cardiovascular**	**6.0 (5.0–7.1)**	**6.5 (5.0–7.9)**	**8.4** * **(6.8–10.0)**	**10.9** * **(9.4–12.4)**	**12.5** * **(11.2–13.7)**	**10.7** * **(9.3–12.1)**
CHF	1.1 (0.7–1.4)	1.9* (1.0–2.8)	2.4* (1.7–3.1)	2.9* (2.0–3.7)	4.3* (3.7–4.8)	3.7* (2.9–4.4)
CHD	3.1 (2.2–4.1)	2.6 (1.7–3.6)	3.8 (2.9–4.8)	4.1 (3.0–5.2)	4.4* (3.5–5.3)	3.3 (2.5–4.0)
Stroke	1.8 (1.4–2.3)	1.9 (1.3–2.6)	2.7* (1.8–3.5)	3.5* (2.8–4.3)	4.6* (4.0–5.1)	4.6* (3.7–5.4)
Angina pectoris	1.3 (0.9–1.8)	1.9 (1.1–2.7)	1.7 (1.0–2.4)	2.9* (2.1–3.8)	2.9* (2.3–3.4)	2.7* (2.0–3.4)
MI	2.4 (1.6–3.2)	2.4 (1.5–3.4)	2.7 (1.8–3.6)	3.5 (2.6–4.5)	5.0* (4.2–5.9)	4.1* (3.3–4.9)
**Endocrine and metabolic**	**10.5 (9.3–11.8)**	**12.4 (10.1–14.7)**	**12.8** * **(11.1–14.4)**	**15.2** * **(13.0–17.4)**	**16.5** * **(15.0–17.9)**	**17.3** * **(15.8–18.8)**
Diabetes^d^	8.0 (6.7–9.2)	9.8 (7.8–11.8)	10.0 (8.5–11.6)	12.0* (10.0–14.0)	11.9* (10.8–12.9)	12.5* (11.2–13.8)
Renal disease	1.9 (1.3–2.4)	2.3 (1.3–3.2)	2.7 (1.8–3.5)	3.1* (2.3–3.9)	4.2* (3.6–4.9)	3.6* (2.8–4.5)
Liver disease (current)^e^	1.4 (0.9–1.9)	1.8 (1.0–2.6)	1.6 (1.0–2.3)	2.4* (1.6–3.2)	2.5* (2.0–3.1)	3.7* (2.5–5.0)

*Note:* Diagnosed estimates include individuals who reported having a diagnosis of a RF in the interview component. Hispanic group defined in NHANES as Mexican American or other Hispanic. PIR calculated by NHANES by dividing family (or individual) income by the US Health and Human Services poverty guidelines relevant to the survey year; a ratio < 1 represents family income below the poverty level. Other race and ethnicity/multi‐racial results and missing PIR results are not presented. See Tables S2 (by race and ethnicity) and S3 (by PIR) for stratifications by age group.

Abbreviations: CHD, coronary heart disease; CHF, congestive heart failure; CI, confidence interval; COPD, chronic obstructive pulmonary disease; MI, myocardial infarction; NHANES, National Health and Nutrition Examination Survey; PIR, poverty income ratio; RF, risk factor; RSV, respiratory syncytial virus; SP, sample person; US, United States.

^a^ Summary measures of RFs include those measurable via NHANES: COPD, asthma (current), CHF, CHD, stroke, angina pectoris, MI, diabetes, renal disease, and/or liver disease (current).

^b^ Defined as a “yes” response to any of the following questionnaire items: MCQ160o (Has a doctor or other health professional ever told [you/SP] that [you/s/he] had COPD? 2013–2014 and 2015–2016 only) or MCQ170k (Do you still have chronic bronchitis? 2011–2012, 2013–2014, and 2015–2016 only), MCQ160g (Has a doctor or other health professional ever told [you/SP] that [you/s/he] had emphysema? 2011–2012, 2013–2014, and 2015–2016 only), or MCQ160p ([Have you/Has SP] ever been told by a doctor or other health professional that [you/he/she] had chronic obstructive pulmonary disease or COPD, emphysema, or chronic bronchitis? 2017–March 2020 only).

^c^ Only asked among respondents who answered “yes” to having ever received a diagnosis of asthma. Respondents who did not report ever receiving a diagnosis of asthma are included in the “no” category.

^d^ Respondents who reported having borderline diabetes are included in the “no” category.

^e^ Only asked among respondents who answered “yes” to having ever received a diagnosis of liver disease. Respondents who did not report ever receiving a diagnosis of liver disease are included in the “no” category.

* Statistically significant (*p* < 0.05) based on pairwise chi‐square analysis on 2 × 2 tables comparing the proportion of respondents with each RF in the respective group to the reference group (i.e., non‐Hispanic White or PIR ≥ 5).

**TABLE 2:** Percentage of adults aged ≥ 20 years with ≥ 1 diagnosed RF for severe RSV disease, by age group.

	20–49 years (*N* = 125,255,765)	50–59 years (*N* = 42,750,712)	≥ 60 years (*N* = 65,095,825)
RFs, % (95% CI)	Overall		
**≥ 1 RF** ^**a**^	**17.0 (16.1–18.0)**	**31.4** * **(29.2–33.7)**	**46.9** * **(45.0–48.8)**
1 RF	13.8 (13.0–14.5)	20.7* (18.7–22.6)	24.8* (23.2–26.4)
2 RFs	2.4 (2.0–2.8)	6.5* (5.5–7.4)	11.4* (10.4–12.4)
3 RFs	0.5 (0.4–0.7)	2.4* (1.9–3.0)	5.3* (4.7–5.9)
≥ 4 RFs	0.3 (0.2–0.4)	1.9* (1.3–2.5)	5.4* (4.6–6.2)
**≥ 1 RF, % (95% CI)**	**By race and ethnicity**		
Non‐Hispanic White	17.7 (16.3–19.2)	29.9 (26.8–32.9)	44.9 (42.5–47.4)
Hispanic	15.0* (13.5–16.5)	34.1* (30.8–37.3)	50.4* (47.7–53.0)
Non‐Hispanic Black	19.2 (17.4–21.0)	36.9* (33.9–39.8)	54.0* (51.5–56.6)
Non‐Hispanic Asian	10.0* (8.3–11.7)	24.1* (20.8–27.3)	43.6 (40.0–47.3)
**≥ 1 RF, % (95% CI)**	**By PIR**		
≥ 5	13.2 (11.3–15.1)	19.4 (15.7–23.1)	35.4 (31.4–39.4)
4–< 5	15.3 (12.3–18.4)	23.4 (16.9–30.0)	41.0 (34.8–47.2)
3–< 4	14.8 (12.2–17.4)	32.3* (25.4–39.1)	44.2* (38.6–49.8)
2–< 3	17.3* (15.0–19.6)	37.9* (30.3–45.4)	49.5* (45.1–53.9)
1–< 2	18.6* (16.5–20.8)	39.1* (35.3–42.9)	57.6* (54.7–60.5)
< 1	22.7* (20.5–24.9)	49.1* (44.0–54.2)	58.1* (54.2–61.9)

*Note:* Diagnosed estimates include individuals who reported having a diagnosis of a RF in the interview component. Hispanic group defined in NHANES as Mexican American or other Hispanic. PIR calculated by NHANES by dividing family (or individual) income by the US Health and Human Services poverty guidelines relevant to the survey year; a ratio < 1 represents family income below the poverty level. Other race and ethnicity/multi‐racial results and missing PIR results are not presented. See Tables S2 (by race and ethnicity) and S3 (by PIR) for stratification by individual RFs.

Abbreviations: CHD, coronary heart disease; CHF, congestive heart failure; CI, confidence interval; COPD, chronic obstructive pulmonary disease; MI, myocardial infarction; NHANES, National Health and Nutrition Examination Survey; PIR, poverty income ratio; RF, risk factor; RSV, respiratory syncytial virus; US, United States.

^a^ Summary measures of RFs include those measurable via NHANES: COPD, asthma (current), CHF, CHD, stroke, angina pectoris, MI, diabetes, renal disease, and/or liver disease (current).

* Statistically significant (*p* < 0.05) based on pairwise chi‐square analysis on 2 × 2 tables comparing the proportion of respondents with each RF in the respective group to the reference group. For the overall results, age 20–49 years is the reference group. For results by race and ethnicity and by PIR, non‐Hispanic White and PIR ≥ 5 are the reference groups (within each age group).

Significant disparities were observed across race and ethnicity among adults aged ≥ 50 years, with Hispanic and non‐Hispanic Black adults having significantly higher prevalence of ≥ 1 RF vs. non‐Hispanic White adults in those aged 50–59 years (34.1% and 36.9%, respectively, vs. 29.9%) and in those aged ≥ 60 years (50.4% and 54.0%, respectively, vs. 44.9%; Table S2). Compared with non‐Hispanic White adults aged ≥ 20 years, non‐Hispanic Black adults had a significantly higher prevalence of asthma, CHF, stroke, and diabetes (Table 1).

Overall, adults in the lowest PIR category (< 1) had a significantly higher prevalence of ≥ 1 diagnosed RF than those with PIR ≥ 5 (33.8% vs. 21.0%, respectively); the same was true for ≥ 1 pulmonary‐related RF (17.6% vs. 8.8%, respectively), ≥ 1 cardiovascular‐related RF (10.7% vs. 6.0%, respectively), and ≥ 1 endocrine/metabolic‐related RF (17.3% vs. 10.5%, respectively; Table 1). Disparities were more pronounced with increasing age, with adults with PIR < 4 having significantly higher prevalence of ≥ 1 RF vs. adults with PIR ≥ 5 among those aged 50–59 years and ≥ 60 years (Table 2; Table S3).

### 3.3 Prevalence of Undiagnosed Endocrine/Metabolic RFs for Severe RSV Disease

**FIGURE 1:**
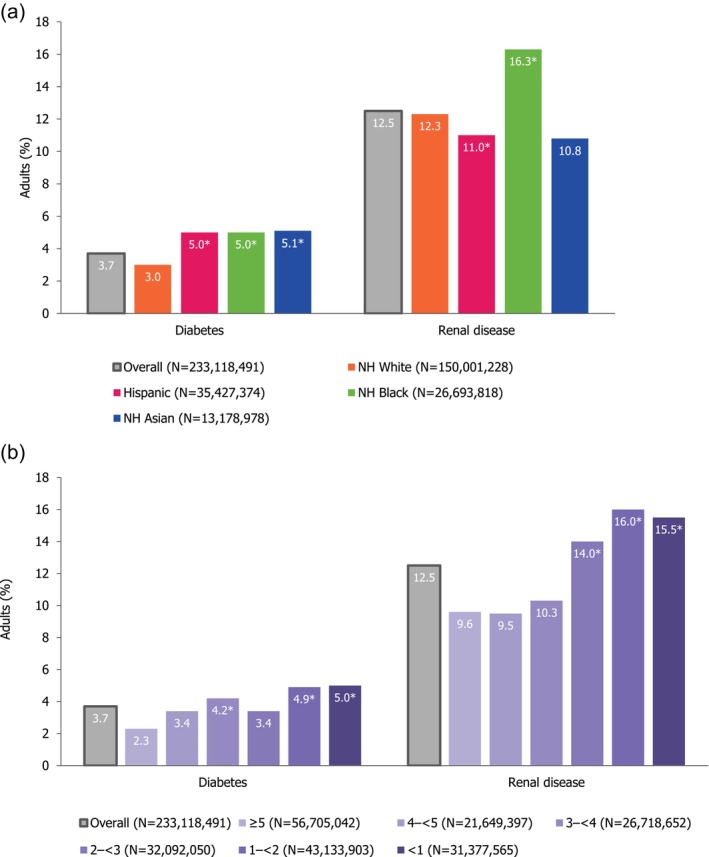
Percentage of adults aged ≥ 20 years with undiagnosed diabetes and renal disease. (a) By race and ethnicity. (b) By PIR. Undiagnosed diabetes defined as no self‐reported diagnosis in the interview component and plasma fasting glucose > 126 mg/dL (≥ 7.0 mmol/L), or HbA1C > 6.5%, or a 2‐h oral glucose tolerance test result ≥ 200 mg/dL. The 2‐h oral glucose tolerance test is not available in 2017–March 2020. Undiagnosed renal disease defined as no self‐reported diagnosis in the interview component and eGFR < 60 mg/dL (CKD‐EPI formula) or urine albumin creatinine ratio ≥ 30 mg/g. Hispanic group defined in NHANES as Mexican American or other Hispanic. PIR calculated by NHANES by dividing family (or individual) income by the US Health and Human Services poverty guidelines relevant to the survey year; a ratio < 1 represents family income below poverty level. Other race and ethnicity/multi‐racial results and missing PIR results not presented. See Table S4 for stratification by age group. *Statistically significant (*p* < 0.05) based on pairwise chi‐square analysis on 2 × 2 tables comparing proportion of respondents with each RF in respective group to the reference group (i.e., non‐Hispanic White or PIR ≥ 5). Abbreviations: CKD‐EPI, Chronic Kidney Disease Epidemiology Collaboration; eGFR, estimated glomerular filtration rate; HbA1C, hemoglobin A1C; NH, non‐Hispanic; NHANES, National Health and Nutrition Examination Survey; PIR, poverty income ratio; US, United States.

Adults with undiagnosed (i.e., diagnosis not self‐reported in the interview component) diabetes and renal disease were identified through laboratory tests (Figure 1). Among adults aged ≥ 20 years, 3.7% (95% CI: 3.3%–4.0%) were found to have undiagnosed diabetes through laboratory testing, compared with the reported diagnosed prevalence of 10.6% (10.1%–11.1%). Laboratory results also revealed that 12.5% (11.8%–13.1%) of adults had undiagnosed renal disease, compared with the reported diagnosed prevalence of 3.0% (2.7%–3.3%).

Compared with non‐Hispanic White adults, the prevalence of undiagnosed diabetes was significantly higher among Hispanic, non‐Hispanic Black, and non‐Hispanic Asian adults (5.0%–5.1% vs. 3.0%), and the prevalence of undiagnosed renal disease was significantly higher among non‐Hispanic Black adults (16.3% vs. 12.3%, respectively). Compared with adults with PIR ≥ 5, the prevalence of undiagnosed diabetes was significantly higher among adults with PIR < 2 (4.9%–5.0% vs. 2.3%), as was the prevalence of undiagnosed renal disease among those with PIR < 3 (14.0%–16.0% vs. 9.6%).

Similar to the trend observed for diagnosed RFs, the prevalence of undiagnosed diabetes and renal disease both increased with age, with differences across racial and ethnic groups and PIR being more pronounced with increasing age (Table S4). For example, among those aged ≥ 60 years, undiagnosed diabetes and renal disease were identified in 6.6% (5.8%–7.4%) and 25.6% (24.2%–27.0%) of adults, respectively.

### 3.4 Combined Prevalence of Diagnosed RFs for Severe RSV Disease and Undiagnosed Diabetes and Renal Disease

The overall combined prevalence of ≥ 1 RF, including diagnoses reported in the interview and undiagnosed diabetes and/or renal disease confirmed via laboratory testing, among adults aged ≥ 20 years was 35.4% (95% CI: 34.2%–36.5%). Prevalence significantly increased with age (20–49 years: 22.4% [21.4%–23.5%]; 50–59 years: 38.1% [35.7%–40.6%]; ≥ 60 years: 58.4% [56.6%–60.2%]; Table S5).

**FIGURE 2:**
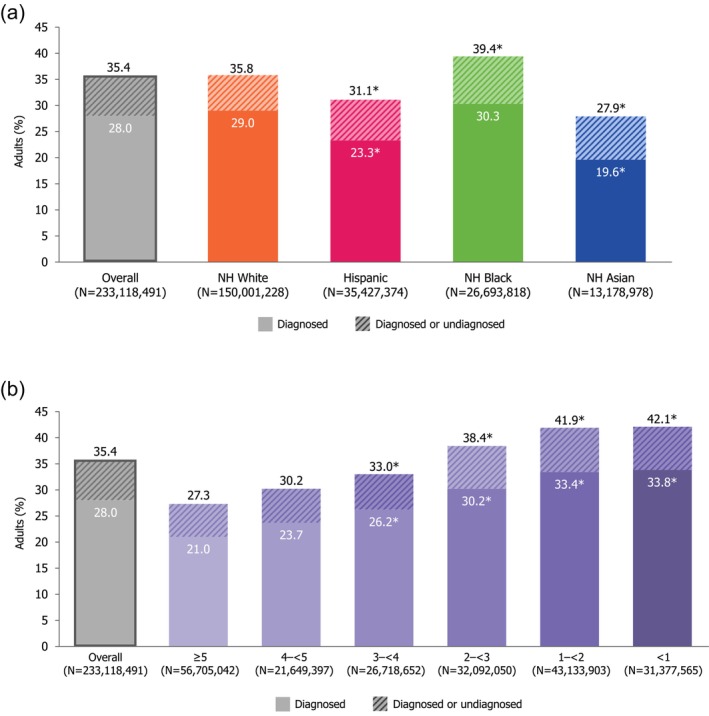
Combined percentage of adults aged ≥ 20 years with ≥ 1 diagnosed RF for severe RSV disease or undiagnosed diabetes or renal disease^a^. (a) By race and ethnicity. (b) By PIR. Hispanic group defined in NHANES as Mexican American or other Hispanic. PIR calculated by NHANES by dividing family (or individual) income by the US Health and Human Services poverty guidelines relevant to the survey year; a ratio < 1 represents family income below poverty level. Other race and ethnicity/multi‐racial results and missing PIR results not presented. See Table S5 for stratification by age group. ^a^Diagnosed estimates include individuals who reported having a diagnosis of a RF in the interview component. Undiagnosed estimates include individuals who reported not having a diagnosis of a RF in the interview component, but the corresponding examination/laboratory component indicates presence of condition. Overall estimates include individuals who reported having received a diagnosis of a given RF in the interview component, individuals who reported not having a diagnosis of a RF in the interview component but the corresponding examination/laboratory component indicates presence of condition, and individuals who did not answer or responded “do not know” to the interview component but the corresponding examination/laboratory component indicates presence of a given RF. Of the 10 included RFs for severe RSV disease measurable via NHANES (COPD, asthma [current], CHF, CHD, stroke, angina pectoris, MI, diabetes, renal disease, and/or liver disease [current]), examination/laboratory tests were only available to determine the presence of diabetes and renal disease. *Statistically significant (*p* < 0.05) based on pairwise chi‐square analysis on 2 × 2 tables comparing proportion of respondents with each RF in respective group to the reference group (i.e., non‐Hispanic White or PIR ≥ 5). Abbreviations: CHD, coronary heart disease; CHF, congestive heart failure; COPD, chronic obstructive pulmonary disease; MI, myocardial infarction; NH, non‐Hispanic; NHANES, National Health and Nutrition Examination Survey; PIR, poverty income ratio; RF, risk factor; RSV, respiratory syncytial virus; US, United States.

Similar to analyses limited to diagnosed RFs, the overall combined prevalence of ≥ 1 RF among adults aged ≥ 20 years was highest among those with the lowest PIR of < 1 (42.1% [40.1%–44.2%]) and among non‐Hispanic Black adults (39.4% [37.6%–41.2%]; Figure 2).

### 3.5 Mean Age at Diagnosis of RFs for Severe RSV Disease

Among adults aged ≥ 20 years with ≥ 1 RF, mean age at self‐reported diagnosis of ≥ 1 RF was 40.1 (95% CI: 39.3–41.0) years (Table 3). Mean age was lowest among adults with ≥ 1 diagnosed pulmonary RF, mainly attributable to the early average age of asthma diagnosis at 24.1 (22.7–25.4) years, and highest among adults with cardiovascular RFs, peaking at 57.1 (55.6–58.6) years for CHF.

**TABLE 3:** Mean age at diagnosis, among adults aged ≥ 20 years with ≥ 1 diagnosed RF for severe RSV disease.

Mean age at RF diagnosis, years (95% CI)	Overall and by race and ethnicity				
	Overall (*N* = 58,999,617)	Non‐Hispanic White (*N* = 38,994,125)	Hispanic (*N* = 7,489,193)	Non‐Hispanic Black (*N* = 7,420,066)	Non‐Hispanic Asian (*N* = 2,386,308)
**≥ 1 RF** ^**a**^	**40.1 (39.3–41.0)**	**41.2 (40.1–42.4)**	**36.9** * **(35.6–38.1)**	**36.8** * **(35.5–38.1)**	**43.7** * **(41.8–45.7)**
1 RF	38.6 (37.6–39.7)	39.4 (37.8–40.9)	36.0* (34.7–37.4)	36.0* (34.2–37.8)	42.8* (40.7–44.9)
2 RFs	43.0 (41.5–44.4)	44.9 (42.8–46.9)	38.9* (36.3–41.5)	38.4* (36.0–40.8)	45.7 (41.5–49.8)
3 RFs	44.2 (42.3–46.2)	45.7 (43.3–48.0)	38.4* (35.1–41.7)	38.8* (35.7–41.9)	48.5 (43.2–53.9)
≥ 4 RFs	41.1 (38.9–43.3)	41.8 (39.0–44.5)	40.8 (36.4–45.1)	37.7 (33.5–41.9)	45.6 (37.6–53.6)
**Pulmonary and cardiovascular**	**38.5 (37.5–39.6)**	**40.2 (38.7–41.7)**	**32.8** * **(30.8–34.7)**	**33.9** * **(32.4–35.4)**	**41.3 (38.1–44.5)**
**Pulmonary**	**26.8 (25.5–28.1)**	**28.1 (26.3–30.0)**	**23.2** * **(21.0–25.4)**	**22.5** * **(21.0–24.1)**	**29.4 (25.6–33.3)**
COPD^b^	43.2 (41.3–45.2)	44.2 (41.7–46.6)	41.9 (37.8–46.1)	35.5* (32.3–38.7)	46.5 (36.8–56.2)
Asthma (current)^c^	24.1 (22.7–25.4)	24.8 (22.9–26.7)	22.3 (20.0–24.6)	21.2* (19.6–22.8)	29.3* (25.4–33.1)
**Cardiovascular**	**54.1 (53.4–54.9)**	**55.6 (54.6–56.5)**	**48.1** * **(46.1–50.2)**	**50.4** * **(49.0–51.8)**	**55.4 (53.0–57.8)**
CHF	57.1 (55.6–58.6)	59.2 (57.3–61.1)	50.4* (47.1–53.7)	51.8* (49.3–54.4)	54.3 (47.9–60.8)
CHD	57.0 (55.9–58.0)	57.9 (56.6–59.3)	51.0* (47.4–54.7)	53.2* (50.9–55.5)	57.0 (53.8–60.2)
Stroke	55.4 (54.3–56.6)	57.0 (55.3–58.6)	50.0* (46.7–53.2)	52.1* (50.3–53.8)	58.5 (53.9–63.1)
Angina pectoris	51.6 (50.2–53.0)	52.7 (51.0–54.4)	45.0* (41.3–48.7)	46.9* (44.2–49.7)	55.4 (51.4–59.4)
MI	54.9 (54.0–55.8)	55.9 (54.7–57.1)	51.9* (49.1–54.7)	50.6* (47.9–53.4)	53.4 (49.3–57.6)
**Endocrine and metabolic**	**47.4 (46.6−48.2)**	**48.4 (47.3−49.6)**	**44.1** * **(42.8−45.4)**	**46.0** * **(44.8−47.1)**	**48.5 (46.5–50.4)**
Diabetes	48.3 (47.5−49.0)	49.5 (48.3−50.7)	44.5* (43.2−45.9)	46.2* (45.0−47.3)	50.4 (48.5–52.3)
Renal disease^d^	N/A	N/A	N/A	N/A	N/A
Liver disease (current)^e^	44.6 (42.9–46.3)	45.0 (42.6−47.4)	43.9 (41.4–46.3)	46.2 (42.2–50.2)	41.4 (37.3–45.4)

Mean age at RF diagnosis, years (95% CI)	By PIR					
	≥ 5 (*N* = 10,841,666)	4–< 5 (*N* = 4,840,077)	3–< 4 (*N* = 6,418,387)	2–< 3 (*N* = 8,818,228)	1–< 2 (*N* = 12,846,534)	< 1 (*N* = 9,548,521)
**≥ 1 RF** ^**a**^	**40.8 (38.6–43.1)**	**38.3 (35.9–40.7)**	**41.9 (39.7–44.1)**	**41.0 (39.0–42.9)**	**41.9 (40.6–43.2)**	**35.1** * **(33.6–36.6)**
1 RF	38.9 (36.4–41.4)	36.6 (33.1–40.0)	40.2 (37.6–42.9)	39.1 (36.8–41.4)	40.3 (38.7–41.9)	34.0* (32.1–35.9)
2 RFs	45.3 (41.3–49.3)	39.2 (33.1–45.3)	47.0 (43.7–50.4)	44.7 (40.6–48.7)	43.8 (40.7–46.8)	37.5* (34.9–40.2)
3 RFs	48.1 (42.9–53.3)	45.5 (37.9–53.1)	47.4 (41.9–52.9)	44.3 (39.0–49.7)	47.5 (44.2–50.7)	35.2* (31.9–38.4)
≥ 4 RFs	44.5 (36.3–52.6)	46.1 (38.2–54.0)	38.7 (29.5–47.9)	42.5 (35.7–49.4)	42.3 (38.8–45.8)	36.5 (32.2–40.7)
**Pulmonary and cardiovascular**	**39.2 (36.3–42.2)**	**35.2 (31.9–38.6)**	**40.4 (37.3–43.5)**	**39.2 (36.5–42.0)**	**40.9 (39.4–42.4)**	**33.2** * **(31.6–34.8)**
**Pulmonary**	**25.5 (22.5–28.5)**	**24.3 (20.8–27.9)**	**28.5 (23.9–33.1)**	**25.6 (22.1–29.0)**	**29.6** * **(27.4–31.7)**	**25.8 (23.6–27.9)**
COPD^b^	39.3 (32.1–46.5)	46.7 (38.3–55.0)	51.8* (43.2–60.3)	44.0 (36.5–51.5)	43.9 (41.0–46.8)	38.3 (35.2–41.4)
Asthma (current)^c^	24.4 (21.4–27.5)	22.0 (18.1–25.9)	25.0 (20.5–29.4)	22.7 (19.4–26.1)	26.0 (24.1–27.9)	23.2 (20.9–25.5)
**Cardiovascular**	**57.1 (55.3–59.0)**	**54.8 (50.8–58.7)**	**55.3 (53.0–57.7)**	**55.0 (53.5–56.5)**	**54.4** * **(52.8–55.9)**	**48.5** * **(47.1–49.9)**
CHF	60.4 (56.3–64.5)	56.9 (50.2–63.7)	57.2 (53.6–60.7)	60.5 (57.6–63.4)	57.9 (55.4–60.4)	51.4* (48.8–53.9)
CHD	57.5 (55.7–59.4)	55.6 (50.2–61.0)	60.7* (58.2–63.1)	58.3 (55.7–60.8)	58.1 (56.0–60.1)	52.1* (50.5–53.7)
Stroke	59.1 (55.1–63.0)	59.7 (54.9–64.4)	55.7 (51.4–60.0)	55.8 (51.5–60.2)	56.3 (53.3–59.3)	49.7* (47.2–52.1)
Angina pectoris	54.1 (49.4–58.7)	51.6 (46.8–56.4)	48.3 (42.5–54.1)	51.9 (48.8–55.1)	53.3 (50.1–56.5)	46.5* (43.3–49.7)
MI	57.3 (54.7–59.9)	55.5 (52.6–58.5)	54.8 (51.1–58.5)	54.7 (51.7–57.7)	56.9 (54.9–58.8)	49.5* (47.4–51.5)
**Endocrine and metabolic**	**47.3 (45.5–49.0)**	**48.8 (46.7–50.8)**	**47.5 (45.6–49.5)**	**49.4 (47.9–51.0)**	**48.7 (47.1–50.2)**	**43.5** * **(41.6–45.4)**
Diabetes	47.7 (45.8–49.7)	50.4 (48.3–52.5)	47.5 (45.6–49.4)	50.3 (48.5–52.0)	49.7 (48.1–51.2)	44.1* (42.5–45.8)
Renal disease^d^	N/A	N/A	N/A	N/A	N/A	N/A
Liver disease (current)^e^	45.7 (41.6–49.8)	42.5 (37.4–47.6)	49.0 (43.7–54.3)	45.4 (41.2–49.7)	45.0 (40.9–49.1)	43.0 (37.9–48.0)

*Note:* Age at diagnosis for diagnosed estimates includes age at self‐reported diagnosis among individuals who reported having a diagnosis of a RF in the interview component. Hispanic group defined in NHANES as Mexican American or other Hispanic. PIR calculated by NHANES by dividing family (or individual) income by the US Health and Human Services poverty guidelines relevant to the survey year; a ratio < 1 represents family income below the poverty level. Other race and ethnicity/multi‐racial results and missing PIR results are not presented. See Table S6 for mean age at identification of undiagnosed RFs (i.e., diabetes and renal disease).

Abbreviations: CHD, coronary heart disease; CHF, congestive heart failure; CI, confidence interval; COPD, chronic obstructive pulmonary disease; MI, myocardial infarction; NHANES, National Health and Nutrition Examination Survey; PIR, poverty income ratio; RF, risk factor; RSV, respiratory syncytial virus; US, United States.

^a^ Summary measures of RFs include those measurable via NHANES: COPD, asthma (current), CHF, CHD, stroke, angina pectoris, MI, diabetes, and/or liver disease (current). The earliest age at diagnosis of any of the respective diagnoses for each respondent is considered.

^b^ There is no age at diagnosis question for the COPD question itself, only for chronic bronchitis and emphysema individually; however, they are not asked individually in 2017–March 2020. Age at diagnosis is considered the earliest diagnosis of current chronic bronchitis or emphysema. Only respondents who report current chronic bronchitis diagnosis and/or emphysema diagnosis are included.

^c^ Presence of current asthma was asked among respondents who answered “yes” to having ever received a diagnosis of asthma. The age presented is the age at first asthma diagnosis among those respondents who report currently having asthma.

^d^ Age at self‐reported diagnosis is not available for renal disease.

^e^ Presence of current liver disease was asked among respondents who answered “yes” to having ever received a diagnosis of liver disease. The age presented is the age at first liver disease diagnosis among those respondents who report currently having liver disease.

* Statistically significant (*p* < 0.05) based on Wald‐pairwise t‐test analysis on 2 × 2 tables comparing mean age at diagnosis in respective groups to the reference group (i.e., non‐Hispanic White or PIR ≥ 5).

On average, non‐Hispanic Black and Hispanic adults were diagnosed with ≥ 1 RF approximately 4 years earlier than non‐Hispanic White adults (age 36.8 [35.5–38.1] and 36.9 [35.6–38.1] vs. 41.2 [40.1–42.4] years, respectively), and adults with PIR < 1 were diagnosed more than 5 years earlier than adults with PIR ≥ 5 (age 35.1 [33.6–36.6] vs. 40.8 [38.6–43.1] years, respectively).

Mean age at identification of undiagnosed diabetes and renal disease is summarized in Table S6; younger age at identification of undiagnosed RFs was observed among Hispanic and non‐Hispanic Black adults and adults with PIR < 1, consistent with findings for diagnosed RFs.

### 3.6 Diagnosis of RFs for Severe RSV Disease Before Age 50 and 60 Years

Among adults aged ≥ 20 years with ≥ 1 diagnosed RF, 60.0% (95% CI: 58.0%–62.0%) were diagnosed before age 50 years and 80.4% (78.8%–81.9%) were diagnosed before age 60 years. Pulmonary‐related RFs were typically diagnosed before age 50 years, including asthma (84.3% [81.7%–86.8%]) and COPD (54.8% [49.4%–60.2%]). Overall, approximately one in three (34.0% [31.5%–36.6%]) adults with a cardiovascular‐related RF were diagnosed before age 50 years, ranging from 23.9% (19.5%–28.2%) for CHD to 42.2% (36.0%–48.4%) for angina pectoris. A total of 47.6% (45.0%–50.3%) of adults with diabetes and 61.2% (53.7%–68.6%) of adults with liver disease reported receiving their diagnosis before age 50 years.

**FIGURE 3:**
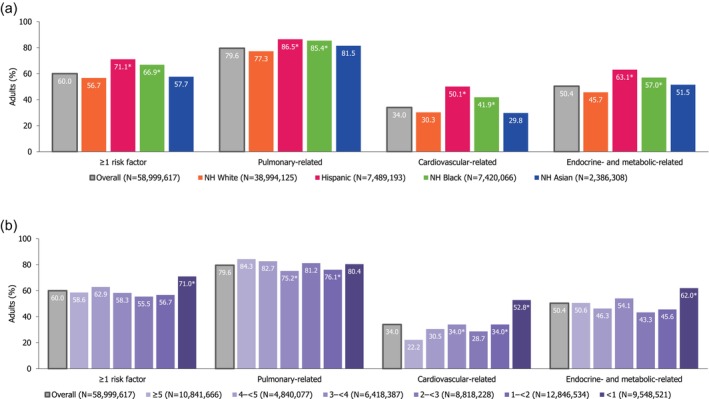
Percentage of adults aged ≥ 20 years with ≥ 1 RF for severe RSV disease, diagnosed before age 50 years^a^. (a) By race and ethnicity. (b) By PIR. Age at diagnosis for diagnosed estimates includes age at self‐reported diagnosis among individuals who reported having a diagnosis of a RF in the interview component. Hispanic group defined in NHANES as Mexican American or other Hispanic. PIR calculated by NHANES by dividing family (or individual) income by the US Health and Human Services poverty guidelines relevant to the survey year; a ratio < 1 represents family income below poverty level. Other race and ethnicity/multi‐racial results and missing PIR results not presented. See Table S7 for stratification by individual RFs. ^a^Summary measures of RFs include those measurable via NHANES: COPD, asthma (current), CHF, CHD, stroke, angina pectoris, MI, diabetes, and/or liver disease (current). Age at self‐reported diagnosis is not available for renal disease. The earliest age at diagnosis of any of the respective diagnoses for each respondent is considered. *Statistically significant (*p* < 0.05) based on pairwise chi‐square analysis on 2 × 2 tables comparing proportion of respondents with each RF in respective group to the reference group (i.e., non‐Hispanic White or PIR ≥ 5). Abbreviations: CHD, coronary heart disease; CHF, congestive heart failure; COPD, chronic obstructive pulmonary disease; MI, myocardial infarction; NH, non‐Hispanic; NHANES, National Health and Nutrition Examination Survey; PIR, poverty income ratio; RF, risk factor; RSV, respiratory syncytial virus; US, United States.

Non‐Hispanic Black and Hispanic adults were significantly more likely to be diagnosed with ≥ 1 RF than non‐Hispanic White adults before age 50 years (66.9% and 71.1% vs. 56.7%, respectively). Across all (pulmonary‐, cardiovascular‐, and endocrine/metabolic‐related) RF categories, proportions of RFs diagnosed before age 50 years were highest among Hispanic adults. Adults with PIR < 1 were significantly more likely than those with PIR ≥ 5 to have been diagnosed with ≥ 1 RF before age 50 years (71.0% vs. 58.6%, respectively; Figure 3; Table S7).

### 3.7 Association of SDOH With RFs for Severe RSV Disease

#### 3.7.1 Presence of ≥ 1 RF for Severe RSV Disease Among Adults Aged ≥ 20 Years

**FIGURE 4:**
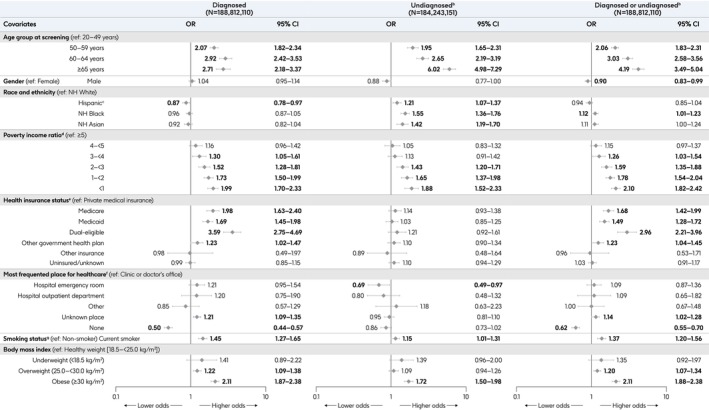
Characteristics associated with the presence of ≥ 1 RF for severe RSV disease, among adults aged ≥ 20 years^a^. Bold values indicate significantly higher or lower odds when compared with the reference group. Highest level of education attained and employment status were not included in the final regression models due to intercorrelation between highest level of education attained and PIR, and between employment status and health insurance status. ^a^≥ 1 RF includes those measurable via NHANES: COPD, asthma (current), CHF, CHD, stroke, angina pectoris, MI, diabetes, renal disease, and/or liver disease (current). ^b^Limited to undiagnosed diabetes and undiagnosed renal disease. Undiagnosed diabetes defined as no self‐reported diagnosis in the interview component and plasma fasting glucose > 126 mg/dL (≥ 7.0 mmol/L), or HbA1C > 6.5%, or a 2‐h oral glucose tolerance test result ≥ 200 mg/dL. The 2‐h oral glucose tolerance test is not available in 2017–March 2020. Undiagnosed renal disease defined as no self‐reported diagnosis in the interview component and eGFR < 60 mg/dL (CKD‐EPI formula) or urine albumin creatinine ratio ≥ 30 mg/g. ^c^Hispanic group defined in NHANES as Mexican American or other Hispanic. ^d^Calculated by NHANES by dividing family (or individual) income by the US Health and Human Services poverty guidelines specific to the survey year; a ratio < 1 represents family income below poverty level. ^e^“Private medical insurance”: reported having private medical insurance AND not having Medicare, Medi‐gap, Medicaid, SCHIP, Military health plan, Indian Health Service, State‐sponsored health plan, or other government insurance. “Medicare”: reported having Medicare AND not having Medicaid. “Medicaid”: reported having Medicaid AND not having Medicare or Medi‐gap. “Dual‐eligible”: reported having Medicare AND Medicaid. “Other government health plan”: reported having Military health plan, SCHIP, Indian Health Service, State‐sponsored health plan, or other government insurance AND are not classified elsewhere. “Other insurance”: reported having some form(s) of insurance AND are not classified elsewhere. “Uninsured/unknown”: reported having no insurance or do not know/refused. ^f^“Clinic or doctor's office”: reported having a most frequent place of healthcare AND the type most often visited as a clinic or health center OR doctor's office or HMO. “Hospital emergency room”: reported having a most frequent place of healthcare AND the type most often visited as an emergency room. “Hospital outpatient department”: reported having a most frequent place of healthcare AND the type most often visited as an outpatient department. “Other”: reported having a most frequent place of healthcare AND the type most often visited as other place or does not go to one place most often. “Unknown place”: reported having a most frequent place of healthcare AND the type most often visited as refused/do not know/missing (includes respondents in the 2017–March 2020 survey cycle who reported having a most frequent place of healthcare because no question was included on the type most often visited). “None”: reported having no routine place to go for healthcare. ^g^Current smoker is defined as having a “yes” response to “do you now smoke cigarettes?” and/or Cotinine laboratory results > 10 ng/mL. Abbreviations: CHD, coronary heart disease; CHF, congestive heart failure; CI, confidence interval; CKD‐EPI, Chronic Kidney Disease Epidemiology Collaboration; COPD, chronic obstructive pulmonary disease; eGFR, estimated glomerular filtration rate; HbA1C, hemoglobin A1C; HMO, health maintenance organization; NH, non‐Hispanic; NHANES, National Health and Nutrition Examination Survey; OR, odds ratio; RF, risk factor; RSV, respiratory syncytial virus; SCHIP, State Children's Health Insurance Program; US, United States.

In multivariable adjusted analyses, age was strongly associated with the presence of ≥ 1 diagnosed or undiagnosed RF, with significantly higher odds in older age groups (Figure 4). Lower PIR was associated with significantly higher odds of having ≥ 1 diagnosed or undiagnosed RF; those with PIR < 1 had 2.1‐fold (95% CI: 1.8–2.4) higher odds than those with PIR ≥ 5. Additionally, being a current smoker and obesity were consistently associated with significantly higher odds of having ≥ 1 diagnosed or undiagnosed RF.

Adults with Medicare, Medicaid, and/or other government health insurance had significantly higher odds of having ≥ 1 diagnosed RF than those with private or commercial insurance. In contrast, adults who reported not having a routine place for healthcare had significantly lower odds of having ≥ 1 diagnosed RF than adults who frequented a clinic or doctor's office. Adults were significantly more likely to have ≥ 1 undiagnosed RF if they were from a racial or ethnic minority group, compared with non‐Hispanic White adults. Meanwhile, those whose routine place for healthcare was the emergency room were significantly less likely to have ≥ 1 undiagnosed RF, compared with adults with a clinic or doctor's office as their routine place for healthcare.

#### 3.7.2 Diagnosis of ≥ 1 RF for Severe RSV Disease Before Age 50 Years

**FIGURE 5:**
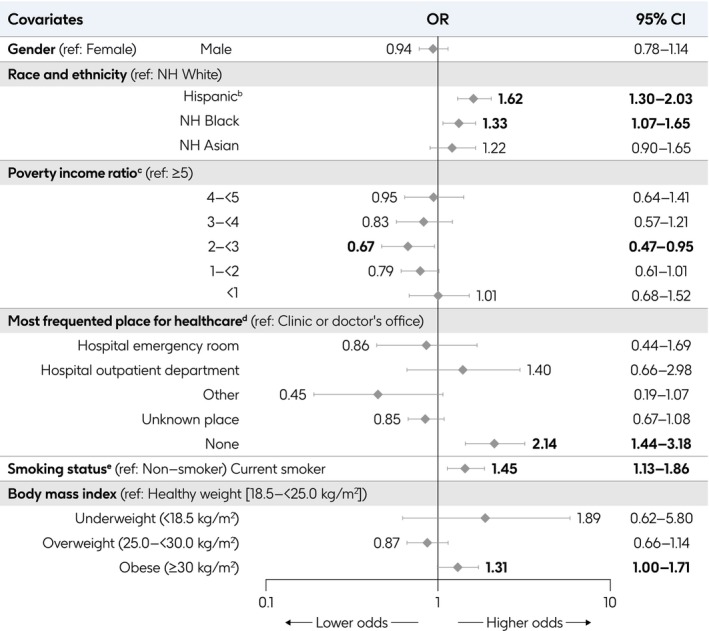
Characteristics associated with the diagnosis of ≥ 1 RF for severe RSV disease before age 50 years, among adults aged ≥ 20 years^a^. Multivariable logistic regression model included *N* = 31,773,835 adults aged ≥ 50 years who were diagnosed with ≥ 1 RF for severe RSV disease. Bold values indicate significantly higher or lower odds when compared with the reference group. Highest level of education attained and employment status were not included in the final regression models due to intercorrelation between the highest level of education attained and PIR, and between employment status and health insurance status. Age group at survey participation was not included in this analysis either as a covariate because a function of all surveys is that each individual must be no older at the time of diagnosis than their age at survey participation; health insurance status was also removed due to its correlation with age. ^a^≥ 1 RF includes those measurable via NHANES: COPD, asthma (current), CHF, CHD, stroke, angina pectoris, MI, diabetes, and/or liver disease (current). Age at self‐reported diagnosis is not available for renal disease. ^b^Hispanic group defined in NHANES as Mexican American or other Hispanic. ^c^Calculated by NHANES by dividing family (or individual) income by the US Health and Human Services poverty guidelines specific to the survey year; a ratio < 1 represents family income below poverty level. ^d^“Clinic or doctor's office”: reported having a most frequent place of healthcare AND the type most often visited as a clinic or health center OR doctor's office or HMO. “Hospital emergency room”: reported having a most frequent place of healthcare AND the type most often visited as an emergency room. “Hospital outpatient department”: reported having a most frequent place of healthcare AND the type most often visited as an outpatient department. “Other”: reported having a most frequent place of healthcare AND the type most often visited as other place or does not go to one place most often. “Unknown place”: reported having a most frequent place of healthcare AND the type most often visited as refused/do not know/missing (includes respondents in 2017–March 2020 survey cycle who reported having a most frequent place of healthcare because no question was included on the type most often visited). “None”: reported having no routine place to go for healthcare. ^e^Current smoker is defined as having a “yes” response to “do you now smoke cigarettes?” and/or Cotinine laboratory results > 10 ng/mL. Abbreviations: CHD, coronary heart disease; CHF, congestive heart failure; CI, confidence interval; COPD, chronic obstructive pulmonary disease; HMO, health maintenance organization; NH, non‐Hispanic; NHANES, National Health and Nutrition Examination Survey; OR, odds ratio; PIR, poverty income ratio; RF, risk factor; RSV, respiratory syncytial virus; US, United States.

Among adults aged ≥ 50 years, non‐Hispanic Black and Hispanic adults had 1.3–1.6‐fold (95% CI: 1.1–1.7 and 1.3–2.0, respectively) higher odds than non‐Hispanic White adults to be diagnosed with ≥ 1 RF by age 50 years. Adults without a routine place for healthcare had 2.1‐fold (1.4–3.2) higher odds of having been diagnosed with ≥ 1 RF before age 50 years than those who frequented a clinic or doctor's office. Being a current smoker was associated with significantly higher odds of being diagnosed with ≥ 1 RF before age 50 years (1.5 [1.1–1.9]), as was obesity (1.3 [1.0–1.7]; Figure 5).

## 4 Discussion

This study used data from several cycles of NHANES to evaluate the prevalence and age at diagnosis of, and characteristics associated with, RFs for severe RSV disease among the US adult population aged ≥ 20 years.

Overall, over one in four (28.0%) adults had ≥ 1 diagnosed RF for severe RSV disease (i.e., CHF, CHD, stroke, angina pectoris, MI, COPD, current asthma, diabetes, current liver disease, and/or renal disease), with prevalence increasing with age (31.4% among adults aged 50–59 years and 46.9% among adults aged ≥ 60 years). This finding likely underestimates the number of adults with RFs, as not all RFs for severe RSV disease are included in NHANES and there are limited corresponding examination and laboratory components to determine the presence of undiagnosed RFs. Two RFs that were ascertained in the relevant NHANES cycles allowed for estimating the prevalence of undiagnosed disease; namely, 3.7% of all adults aged ≥ 20 years had undiagnosed diabetes and 12.5% had undiagnosed renal disease (compared with 10.6% and 3.0% of those with a self‐reported diagnosis in the interview component, respectively). Thus, while the proportion of individuals with undiagnosed diabetes or renal disease differed, the overall prevalence of RFs included in this study is likely even greater than estimated in this analysis.

The prevalence of RFs for severe RSV disease also varied by race and ethnicity and by PIR; prevalence of ≥ 1 overall RF was highest among non‐Hispanic Black adults (39.4%) and adults with PIR < 1 (42.1%). Previous studies have identified similar racial and ethnic disparities, including an analysis of NHANES CY2011–CY2018 data that reported a higher likelihood of cardiovascular and cardiometabolic RFs among Hispanic and non‐Hispanic Black adults compared with non‐Hispanic White and non‐Hispanic Asian adults [27]. Another study using National Health Interview Survey (NHIS) 1999–2018 data identified non‐Hispanic Black individuals persistently displaying the highest prevalence across the study period [9]. An inverse relationship between RF prevalence and PIR that was observed across age groups in our study is consistent with a previous NHANES analysis of CY1999–CY2016 data that reported lower rates of CVD among the highest income group [28]. Likewise, a report using NHIS data from 2011 to 2014 found that the relative difference in diabetes prevalence for individuals classified as middle‐income, near‐poor, and poor was 40.0%, 74.1%, and 100.4% higher than individuals classified as high income, respectively [29]. Evidence from our study builds upon the relationship reported in these previous studies that individuals with the lowest income levels are consistently less healthy, regardless of age and race and ethnicity [30, 31].

Sociodemographic disparities were also observed for age at RF diagnosis. Adults in racial and ethnic minority groups and those of lower income were diagnosed with RFs for severe RSV disease at significantly younger ages than those who were non‐Hispanic White and who had higher income levels, respectively. Of note, non‐Hispanic Black adults were, on average, youngest at time of diagnosis of ≥ 1 pulmonary RF, while Hispanic adults were youngest at time of diagnosis of ≥ 1 cardiovascular or endocrine/metabolic RF. Previous analyses using earlier survey years of NHANES data similarly concluded that age at diagnosis of hypertension, CVD, and diabetes was significantly younger among non‐Hispanic Black and Hispanic adults than non‐Hispanic White adults [13, 14, 15]. The same pattern was found in Caraballo et al. using NHIS 1999–2018 data: self‐reported prevalence of multi‐morbidity among non‐Hispanic Black adults was equivalent to that of Hispanic and non‐Hispanic White adults who were 5 years older [9]. Together, these findings suggest an earlier age at onset of RFs for severe RSV disease among individuals of racial and ethnic minority status and of lower income.

Results of multivariable regression models further reinforce that the burden of RSV disease may be disproportionately shouldered among certain racial and ethnic minority groups and socioeconomically disadvantaged populations. Specifically, increasing age, being non‐Hispanic Black, having lower income, having Medicare, Medicaid, and/or other government health insurance, having no routine place for healthcare, current smoking, and obesity were all significant factors associated with the odds of having ≥ 1 RF for severe RSV disease. Many of these characteristics were also significantly associated with diagnosis of ≥ 1 RF before age 50 years.

Findings from this study underscore the importance of comprehensive RSV vaccine recommendations that allow eligible adults access to vaccination, as well as equitable vaccine implementation programs to protect individuals who are disproportionally at risk of severe RSV disease. Though the CDC's current RSV vaccine recommendation among adults aged 50–74 years is limited to those who are at increased risk of RSV‐LRTD [7], available evidence regarding the impact of such conditional recommendations suggests that vaccine uptake may be suboptimal due to mixed views and understanding of relevant recommendations [32, 33, 34]. Additionally, as this study identified, many adults aged 50–74 years may have undiagnosed diabetes or renal disease, conditions which may qualify them for RSV vaccination as outlined by current ACIP recommendations [35]. These adults, and their healthcare providers, may thus be unaware that they qualify for vaccination, and as a result vulnerable adults remain unprotected against severe RSV disease. Enhancing RF diagnosis in clinical practice may help to more effectively identify at‐risk adults eligible for RSV vaccination. Given that RFs for severe RSV disease are also considered to be risk conditions for other vaccine recommendations, diagnosis of these conditions is key to ensuring that adults are up‐to‐date on vaccinations against vaccine‐preventable diseases more broadly.

The current RSV ACIP vaccine recommendations for adults aged ≥ 50 years may also introduce disparities in RSV vaccination if adults with RFs for severe RSV disease at earlier ages are not recommended for vaccination, despite vaccines being approved for younger adults [4, 5, 6, 35]. Among adults aged ≥ 20 years with ≥ 1 diagnosed RF, our analysis found that almost two in three adults (60.0%) already had ≥ 1 diagnosed RF by age 50 years, with even higher proportions observed among adults who were Hispanic, non‐Hispanic Black, or with lower incomes. Additionally, over three in four adults (80.4%) had ≥ 1 RF diagnosed already by age 60 years. Ultimately, future RSV vaccination recommendations that are universal for all older adults, as well as include younger adults at increased risk of severe RSV disease for which RSV vaccines are approved, may help to protect adults who are disproportionally at risk of severe RSV disease.

Despite the large number of adults aged ≥ 50 years who have ≥ 1 RF and thus qualify for RSV vaccination per the current recommendations [7], vaccine uptake remains suboptimal, with only 16.4% of adults aged ≥ 60 years vaccinated between August 2023 and February 2025 [8]. Disparities in RSV vaccine uptake have also been observed across population subgroups, with markedly lower vaccine uptake among adults of racial and ethnic minority and lower income groups [8, 36]. Considering the high prevalence of RFs for severe RSV disease, younger age of onset for these RFs, and lower vaccine uptake among racial and ethnic minority or socioeconomically disadvantaged adults, these individuals represent key populations who may benefit most from strategies to increase RSV vaccination access and uptake.

It is important to conduct further research to better understand disparities in RSV prevalence, as well as severe RSV‐related outcomes (including hospitalization and death), among subgroups defined by various SDOH. More research is also needed to better understand RSV vaccine outreach and implementation across these subgroups, in addition to opportunities to equitably improve vaccine uptake across the eligible US adult population and ultimately reduce RSV disease burden.

### 4.1 Limitations

The possibility of responder bias was a limitation of the study, as the accuracy of respondents' self‐reported responses could not be verified without medical record confirmation (e.g., self‐report may miss RFs and capture RFs that are not truly present). In addition, the cross‐sectional nature of these data did not allow for derivation of causal relationships or an analysis of changes over time.

Not all RFs for severe RSV disease were captured by data available in the NHANES interview component (e.g., immunocompromised status [including solid organ transplants], interstitial lung disease); others were only asked in certain years (e.g., age at diagnosis of chronic bronchitis/emphysema was not asked in the CY2017–March 2020 cycle) or the wording of the question changed over cycles (e.g., COPD diagnosis). Additionally, not all RFs from the interview component had a corresponding examination/laboratory test (e.g., spirometry), limiting assessment of undiagnosed RFs of interest. Accordingly, the prevalence of RFs may be underestimated, and disparities in RF prevalence may be greater than what is reported herein (given that RFs are disproportionately underdiagnosed in racial and ethnic minority and lower income adults).

Regarding diabetes and renal disease, it is also possible that the diagnostic methods used by NHANES are not sufficiently specific to optimally reflect disease incidence. For example, while one diagnostic criterion for diabetes defined by NHANES was plasma fasting glucose > 126 mg/dL (≥ 7.0 mmol/L), this parameter alone may not fully confirm disease presence and may not be as specific of a measure as HbA1C if it is not measured at more than one time point [19]. Of those classified as having diabetes as per laboratory results in the present study, 57.7% were identified via HbA1C > 6.5%. NHANES classification of diabetes and renal disease may also not directly correlate with corresponding RFs for severe RSV disease as identified by CDC recommendations. Specifically, the ACIP list of RFs includes diabetes complicated by end‐organ damage or requiring treatment with insulin or sodium‐glucose cotransporter‐2 inhibitor, as well as end‐stage renal disease or dependence on renal replacement therapy. Thus, it is possible that not all individuals identified as having these RFs via this analysis would be recommended to receive RSV vaccination under the current CDC recommendations [35].

Whereas age at diagnosis was self‐reported in the interview component, age at diagnosis in the laboratory component was derived from age at screening. Consequently, it is possible that individuals with diagnosed RFs may have lived with these medical conditions for some period of time prior to NHANES screening. Additionally, there was no question for age at diagnosis of COPD; rather, this response was based on chronic bronchitis and emphysema. Finally, though partial CY2019–March 2020 data were combined with data from the previous cycle (CY2017–CY2018) to provide nationally representative 2017–March 2020 pre‐pandemic data, the degree to which this dataset approximates the data which would have been collected without the disruption attributable to COVID‐19 is unknown.

## 5 Conclusions

Approximately one in four US adults aged ≥ 20 years, one in three adults aged 50–59 years, and one in two adults aged ≥ 60 years had ≥ 1 diagnosed RF for severe RSV disease, with almost two in three adults with ≥ 1 RF being diagnosed before age 50 years. The prevalence of RFs, both diagnosed and undiagnosed, varied significantly by sociodemographic characteristics, with higher prevalence among those aged ≥ 50 years, as well as in non‐Hispanic Black or Hispanic adults and those with lower income. Future RSV vaccination recommendations that are universal for older adults, as well as recommendations for younger age groups at increased risk of RSV‐LRTD, may reduce disparities in vaccine access and ultimately in RSV disease burden.

## Author Contributions

**Emily K. Horn:** conceptualization, funding acquisition, investigation, methodology, resources, supervision, validation, visualization, writing – review and editing, writing – original draft. **David Singer:** conceptualization, funding acquisition, writing – original draft, methodology, investigation, resources, supervision, validation, visualization, writing – review and editing. **Alison Booth:** conceptualization, investigation, methodology, project administration, resources, supervision, validation, visualization, writing – original draft. **Hai Nguyen:** investigation, project administration, resources, validation, visualization, writing – review and editing, methodology. **Cynthia Saiontz‐Martinez:** formal analysis, data curation, investigation, resources, software, validation, visualization, writing – review and editing, writing – original draft. **Ariel Berger:** conceptualization, data curation, formal analysis, investigation, methodology, resources, supervision, validation, visualization, writing – review and editing, writing – original draft, project administration.

## Conflicts of Interest

**Emily K. Horn** and **David Singer** are employed by GSK and hold financial equities in GSK. **Alison Booth**, **Hai Nguyen, Cynthia Saiontz‐Martinez**, and **Ariel Berger** are employees of Evidera PPD, which received funding from GSK to conduct this study.

## Supporting information


**Table S1:** Demographic characteristics of the weighted study population in NHANES cycles 2011–March 2020, by survey component available.
**Table S2:** Percentage of adults aged ≥ 20 years with diagnosed RFs for severe RSV disease, by race and ethnicity and age group.
**Table S3:** Percentage of adults aged ≥ 20 years with diagnosed RFs for severe RSV disease, by PIR and age group.
**Table S4:** Percentage of adults aged ≥ 20 years with undiagnosed diabetes and renal disease.
**Table S5:** Combined percentage of adults aged ≥ 20 years with ≥ 1 diagnosed RF for severe RSV disease or undiagnosed diabetes or renal disease^a^.
**Table S6:** Mean age at identification of undiagnosed diabetes and renal disease, among adults aged ≥ 20 years with ≥ 1 undiagnosed RF for severe RSV disease.
**Table S7:** Percentage of adults aged ≥ 20 years with ≥ 1 RF for severe RSV disease diagnosed before age 50 years, stratified by individual RF.

## Data Availability

For requests for access to anonymized subject level data, please contact corresponding author.

## Appendix A NHANES Survey Questions

**Table:** TABLE A1    |    Risk factors for severe RSV disease that were capturable based on information obtained via the questionnaire component of NHANES.

Risk factor	Variable			
	Name	Description	Definition	Availability
Pulmonary				
COPD (COPD, emphysema, or current chronic bronchitis)	MCQ160o	Has a doctor or other health professional ever told [you/SP] that [you/s/he] had COPD?	Binary (COPD present/absent) Present: yes to MCQ160o, or MCQ170k, or MCQ160g, or MCQ160p	2013–2014 2015–2016
	MCQ160p	[Have you/Has SP] ever been told by a doctor or other health professional that [you/he/she] had chronic obstructive pulmonary disease or COPD, emphysema, or chronic bronchitis?		2017–March 2020
	MCQ170k	Do you still have chronic bronchitis?		2011–2012 2013–2014 2015–2016
	MCQ160g	Has a doctor or other health professional ever told [you/SP] that [you/s/he] had emphysema (emph‐phi‐see‐ma)?		2013–2014 2015–2016
Asthma (current)	MCQ035	[Do you/Does SP] still have asthma?	Yes to MCQ010^a^, and Yes to MCQ035 Note: Respondents who did not report ever receiving a diagnosis of asthma are included in the “no” category.	All cycles
Cardiovascular				
Congestive heart failure	MCQ160b	Has a doctor or other health professional ever told [you/SP] that [you/s/he] had congestive heart failure?	Binary (congestive heart failure present/absent)	All cycles
Coronary heart disease	MCQ160c	Has a doctor or other health professional ever told [you/SP] that [you/s/he] had coronary (kor‐o‐nare‐ee) heart disease?	Binary (coronary heart disease present/absent)	All cycles
Stroke	MCQ160f	Has a doctor or other health professional ever told [you/SP] that [you/s/he] had a stroke?	Binary (stroke present/absent)	All cycles
Angina pectoris	MCQ160d	Has a doctor or other health professional ever told [you/SP] that [you/s/he] had angina (an‐gi‐na), also called angina pectoris?	Binary (angina pectoris present/absent)	All cycles
Myocardial infarction	MCQ160e	Has a doctor or other health professional ever told [you/SP] that [you/s/he] had a heart attack (also called myocardial infarction (my‐o‐car‐dee‐al in‐fark‐shun))?	Binary (myocardial infarction present/absent)	All cycles
Endocrine and metabolic				
Diabetes	DIQ010	Other than during pregnancy, [have you/has SP]/[Have you/Has SP] ever been told by a doctor or health professional that [you have/[he/she/SP] has] diabetes or sugar diabetes?	Binary (diabetes present/absent) Note: Respondents who reported having borderline diabetes are included in the “no” category.	All cycles
Renal disease	KIQ022	[Have you/Has SP] ever been told by a doctor or other health professional that [you/s/he] had weak or failing kidneys? Do not include kidney stones, bladder infections, or incontinence	Binary (renal disease present/absent)	All cycles
Liver disease (current)	MCQ170l	[Do you/Does SP] still have any kind of liver condition?	Yes to MCQ160l and Yes to MCQ170l Note: Only asked to respondents who answered “yes” to having ever received a diagnosis of liver disease. Respondents who did not report ever receiving a diagnosis of liver disease are included in the “no” category.	All cycles

Abbreviations: COPD, chronic obstructive pulmonary disease; NHANES, National Health and Nutrition Examination Survey; RSV, respiratory syncytial virus; SP, sample person.

^a^ MCQ010: Has a doctor or other health professional ever told [you/SP] that [you have/s/he/SP] has asthma (az‐ma)?

**Table:** TABLE A2    |    Risk factors for severe RSV disease that were capturable based on information obtained via the laboratory component of NHANES.

Risk factor	Variable			
	Name	Description	Definition	Availability
Endocrine and metabolic				
Diabetes^a^	LBDGLUSI	Fasting glucose (mmol/L)	Binary (diabetes present > 126 mg/dL or ≥ 7.0 mmol/L; diabetes absent ≤ 126 mg/dL or < 7.0 mmol/L) (American Diabetes Association, 2022)	All cycles
	LBXGLU	Fasting glucose (mg/dL)		All cycles
	LBXGH	HbA1C (%)	Binary (diabetes present > 6.5%; diabetes absent ≤ 6.5%) (American Diabetes Association, 2022)	All cycles
	LBDGLTSI	2‐h oral glucose tolerance test (mg/dL)	Binary (diabetes present ≥ 200 mg/dL; diabetes absent < 200 mg/dL) (American Diabetes Association) Note: Diabetes defined as plasma fasting glucose > 126 mg/dL (≥ 7.0 mmol/L), or HbA1C > 6.5%, or a 2‐h oral glucose tolerance test result ≥ 200 mg/dL. The 2‐h oral glucose tolerance test is not available in 2017–March 2020.	2011–2012 2013–2014 2015–2016
Renal disease^b^	LBDSCRSI	Creatinine, serum (μmol/L)	Binary (renal disease present eGFR < 60 mg/dL; renal disease absent eGFR ≥ 60 mg/dL) eGFR was derived using CKD‐EPI formula (National Kidney Foundation, 2023)	All cycles
	URDACT	Albumin creatinine ratio (mg/g)	Binary (renal disease present ≥ 30 mg/g; renal disease absent < 30 mg/g) (National Kidney Foundation, 2023)	All cycles

Abbreviations: CKD‐EPI, Chronic Kidney Disease Epidemiology Collaboration; eGFR, estimated glomerular filtration rate; HbA1C, hemoglobin A1C; NHANES, National Health and Nutrition Examination Survey; RSV, respiratory syncytial virus; SP, sample person.

^a^ An individual was considered to have diabetes if any of the laboratory tests suggested presence.

^b^ An individual was considered to have renal disease if either of the laboratory tests suggested presence.

**Table:** TABLE A3    |    NHANES questions related to age at diagnosis of risk factors for severe RSV disease.

Risk factor	Variable			
	Name	Description	Definition	Availability
Chronic bronchitis	MCQ180k	How old [were you/was SP] when [you were/s/he was] first told [you/s/he] had chronic bronchitis?	Continuous (years)	2011–2012 2013–2014 2015–2016
Emphysema	MCQ180g	How old [were you/was SP] when [you were/s/he was] first told [you/s/he] had emphysema?	Continuous (years)	2011–2012 2013–2014 2015–2016
Asthma	MCQ025	How old [were you/was SP] when [you were/s/he was] first told [you/he/she] had asthma (az‐ma)?	Continuous (years)	All cycles
Congestive heart failure	MCQ180b (MCD180b in 2017–March 2020)	How old [were you/was SP] when [you were/s/he was] first told [you/s/he] had congestive heart failure?	Continuous (years)	All cycles
Coronary heart disease	MCQ180c (MCD180c in 2017–March 2020)	How old [were you/was SP] when [you were/s/he was] first told [you/s/he] had coronary heart disease?	Continuous (years)	All cycles
Stroke	MCQ180f (MCD180f in 2017–March 2020)	How old [were you/was SP] when [you were/s/he was] first told [you/s/he] had a stroke?	Continuous (years)	All cycles
Angina pectoris	MCQ180d (MCD180d in 2017–March 2020)	How old [were you/was SP] when [you were/s/he was] first told [you/s/he] had angina, also called angina pectoris?	Continuous (years)	All cycles
Myocardial infarction	MCQ180e (MCD180e in 2017–March 2020)	How old [were you/was SP] when [you were/s/he was] first told [you/s/he] had a heart attack (also called myocardial infarction)?	Continuous (years)	All cycles
Diabetes	DID040	How old [was SP/were you] when a doctor or other health professional first told [you/him/her] that [you/he/she] had diabetes or sugar diabetes?	Continuous (years)	All cycles
Liver disease	MCQ180l (MCD180l in 2017–March 2020)	How old [were you/was SP] when [you were/s/he was] first told [you/s/he] had any kind of liver condition?	Continuous (years)	All cycles

Abbreviations: NHANES, National Health and Nutrition Examination Survey; RSV, respiratory syncytial virus; SP, sample person.
