# Supplementary material for: Prevalence and Age at Diagnosis of Risk Factors for Severe Respiratory Syncytial Virus Disease Among US Adults: An Analysis of 2011–2020 NHANES Data
Source: Influenza Other Respir Viruses. 2025 Nov 30;19(12):e70181. doi: 10.1111/irv.70181 (PMC12981526; doi:10.1111/irv.70181)
Supplement: Supplementary file 1 — Table S1: Demographic characteristics of the weighted study population in NHANES cycles 2011–March 2020, by survey component available. Table S2: Percentage of adults aged ≥ 20 years with diagnosed RFs for severe RSV disease, by race and ethnicity and age group. Table S3: Percentage of adults aged ≥ 20 years with diagnosed RFs for severe RSV disease, by PIR and age group. Table S4: Percentage of adults aged ≥ 20 years with undiagnosed diabetes and renal disease. Table S5: Combined percentage of adults aged ≥ 20 years with ≥ 1 diagnosed RF for severe RSV disease or undiagnosed diabetes or renal diseasea. Table S6: Mean age at identification of undiagnosed diabetes and renal disease, among adults aged ≥ 20 years with ≥ 1 undiagnosed RF for severe RSV disease. Table S7: Percentage of adults aged ≥ 20 years with ≥ 1 RF for severe RSV disease diagnosed before age 50 years, stratified by individual RF. [file IRV-19-e70181-s001.docx]

### Supplementary Table 1. Demographic characteristics of the weighted study population in NHANES cycles 2011–March 2020, by survey component available

| **Characteristic, n (%)** | **Interview (N=233,118,491)** | **Examination (N=221,518,089)** | **Laboratory (all three components; N=213,158,950)** |
| --- | --- | --- | --- |
| **Age group, years** | | | |
| 20–49 | 125,255,765 (53.7) | 119,055,292 (53.7) | 114,028,445 (53.5) |
| 50–59 | 42,750,712 (18.3) | 40,905,912 (18.5) | 39,659,364 (18.6) |
| 60–64 | 19,204,620 (8.2) | 18,493,471 (8.3) | 17,950,595 (8.4) |
| ≥65 | 45,907,395 (19.7) | 43,063,414 (19.4) | 41,520,546 (19.5) |
| **Gender** | | | |
| Male | 112,042,665 (48.1) | 106,078,179 (47.9) | 102,059,166 (47.9) |
| Female | 121,075,826 (51.9) | 115,439,910 (52.1) | 111,099,784 (52.1) |
| **Race and ethnicity** | | | |
| Non-Hispanic White | 150,001,228 (64.3) | 142,469,542 (64.3) | 138,271,398 (64.9) |
| Hispanic^a^ | 35,427,374 (15.2) | 33,684,703 (15.2) | 32,536,015 (15.3) |
| Non-Hispanic Black | 26,693,818 (11.5) | 25,546,758 (11.5) | 23,585,927 (11.1) |
| Non-Hispanic Asian | 13,178,978 (5.7) | 12,368,168 (5.6) | 11,623,698 (5.5) |
| Other (including multi-racial) | 7,817,093 (3.4) | 7,448,918 (3.4) | 7,141,912 (3.4) |
| **PIR^b^** | | | |
| ≥5 | 56,705,042 (24.3) | 54,201,269 (24.5) | 52,668,794 (24.7) |
| 4–<5 | 21,649,397 (9.3) | 20,710,560 (9.3) | 20,161,718 (9.5) |
| 3–<4 | 26,718,652 (11.5) | 25,809,591 (11.7) | 25,070,519 (11.8) |
| 2–<3 | 32,092,050 (13.8) | 30,737,444 (13.9) | 29,712,810 (13.9) |
| 1–<2 | 43,133,903 (18.5) | 41,363,461 (18.7) | 39,507,691 (18.5) |
| <1 | 31,377,565 (13.5) | 29,977,917 (13.5) | 28,417,157 (13.3) |
| Missing | 21,441,882 (9.2) | 18,717,848 (8.4) | 17,620,260 (8.3) |
| **Health insurance status^c^** | | | |
| Private medical insurance | 114,073,772 (48.9) | 108,786,442 (49.1) | 105,229,479 (49.4) |
| Medicare | 42,122,054 (18.1) | 39,616,885 (17.9) | 38,183,114 (17.9) |
| Medicaid | 16,219,932 (7.0) | 15,314,354 (6.9) | 14,419,180 (6.8) |
| Dual-eligible | 5,012,867 (2.2) | 4,769,155 (2.2) | 4,384,514 (2.1) |
| Other government health plan | 17,509,818 (7.5) | 16,870,162 (7.6) | 16,236,815 (7.6) |
| Other insurance | 1,259,757 (0.5) | 1,174,325 (0.5) | 1,117,691 (0.5) |
| Uninsured/unknown | 36,920,292 (15.8) | 34,986,768 (15.8) | 33,588,157 (15.8) |
| **Highest level of education^d^** | | | |
| College graduate or above | 72,842,330 (31.2) | 69,525,503 (31.4) | 67,124,982 (31.5) |
| Some college or Associate’s  degree | 73,973,098 (31.7) | 70,654,785 (31.9) | 68,078,050 (31.9) |
| High school graduate/GED  equivalent | 53,789,741 (23.1) | 50,729,150 (22.9) | 48,870,184 (22.9) |
| Less than high school | 32,326,318 (13.9) | 30,491,443 (13.8) | 28,979,507 (13.6) |
| **General health status^d^** | | | |
| Excellent | 34,165,021 (14.7) | 32,306,602 (14.6) | 31,077,863 (14.6) |
| Very good | 73,609,498 (31.6) | 69,916,275 (31.6) | 67,595,612 (31.7) |
| Good | 82,446,859 (35.4) | 78,747,077 (35.5) | 75,973,807 (35.6) |
| Fair | 35,436,438 (15.2) | 33,567,488 (15.2) | 32,011,613 (15.0) |
| Poor | 7,207,813 (3.1) | 6,755,360 (3.0) | 6,280,274 (2.9) |
| **Most frequented place for healthcare^e^** | | | |
| Clinic or doctor's office | 117,827,491 (50.5) | 113,752,345 (51.4) | 109,770,598 (51.5) |
| Hospital emergency room | 3,920,245 (1.7) | 3,786,983 (1.7) | 3,636,424 (1.7) |
| Hospital outpatient  department | 1,807,773 (0.8) | 1,754,049 (0.8) | 1,653,851 (0.8) |
| Other | 2,874,767 (1.2) | 2,710,466 (1.2) | 2,607,413 (1.2) |
| Unknown place | 69,423,205 (29.8) | 64,325,957 (29.0) | 61,717,864 (29.0) |
| None | 37,265,010 (16.0) | 35,188,289 (15.9) | 33,772,800 (15.8) |
| **Employment status^d^** | | | |
| Working at a job/business  or with a job/business but  not at work | 146,714,824 (62.9) | 139,789,209 (63.1) | 135,363,651 (63.5) |
| Looking for work | 7,259,942 (3.1) | 6,970,763 (3.1) | 6,620,084 (3.1) |
| Not working at a  job/business | 78,981,573 (33.9) | 74,637,433 (33.7) | 71,054,540 (33.3) |
| **Smoking status^f^** | | | |
| Current smoker | 56,834,532 (24.4) | 54,618,118 (24.7) | 52,877,161 (24.8) |
| Non-smoker | 163,329,661 (70.1) | 160,555,854 (72.5) | 158,847,164 (74.5) |
| Missing | 12,954,298 (5.6) | 6,344,117 (2.9) | 1,434,625 (0.7) |
| **BMI** | | | |
| Underweight (<18.5 kg/m^2^) | 3,215,168 (1.4) | 3,215,168 (1.5) | 3,020,393 (1.4) |
| Healthy weight (18.5–<25.0  kg/m^2^) | 58,734,200 (25.2) | 58,734,200 (26.5) | 55,972,669 (26.3) |
| Overweight (25.0–<30.0  kg/m^2^) | 70,894,601 (30.4) | 70,894,601 (32.0) | 68,785,241 (32.3) |
| Obese (≥30 kg/m^2^) | 86,139,940 (37.0) | 86,139,940 (38.9) | 83,236,105 (39.0) |
| Missing | 14,134,583 (6.1) | 2,534,181 (1.1) | 2,144,542 (1.0) |

^a^Hispanic group defined in NHANES as Mexican American or other Hispanic.

^b^PIR calculated by NHANES by dividing family (or individual) income by the US Health and Human Services poverty guidelines relevant to the survey year. A ratio <1 represents family income below poverty level.

^c^“Private medical insurance”: reported having private medical insurance AND not having Medicare, Medi-gap, Medicaid, SCHIP, Military health plan, Indian Health Service, State-sponsored health plan, or other government insurance. “Medicare”: reported having Medicare AND not having Medicaid. “Medicaid”: reported having Medicaid AND not having Medicare or Medi-gap. “Dual-eligible”: reported having Medicare AND Medicaid. “Other government health plan”: reported having Military health plan, SCHIP, Indian Health Service, State-sponsored health plan, or other government insurance AND are not classified elsewhere. “Other insurance”: reported having some form(s) of insurance AND are not classified elsewhere. “Uninsured/unknown”: reported having no insurance or don’t know/refused.

^d^Missing, “don’t know”, or refused responses not shown; therefore, percentages may not sum to 100%.

^e^“Clinic or doctor's office”: reported having a most frequent place of healthcare AND the type most often visited as a clinic or health center OR doctor's office or HMO. “Hospital emergency room”: reported having a most frequent place of healthcare AND the type most often visited as an emergency room. “Hospital outpatient department”: reported having a most frequent place of healthcare AND the type most often visited as an outpatient department. “Other”: reported having a most frequent place of healthcare AND the type most often visited as other place or doesn't go to one place most often. “Unknown place”: reported having a most frequent place of healthcare AND the type most often visited as refused/don’t know/missing (includes respondents in 2017–March 2020 survey cycle who reported having a most frequent place of healthcare because no question was included on the type most often visited). “None”: reported having no routine place to go for healthcare.

^f^Current smoker is defined as having a “yes” response to “do you now smoke cigarettes?” and/or Cotinine laboratory results >10 ng/mL.

Abbreviations: BMI, body mass index; GED, General Education Development; HMO, health maintenance organization; NHANES, National Health and Nutrition Examination Study; PIR, poverty income ratio; SCHIP, State Children’s Health Insurance Program; US, United States.

### Supplementary Table 2. Percentage of adults aged ≥20 years with diagnosed RFs for severe RSV disease, by race and ethnicity and age group

| RFs, % (95% CI) | **20–49 years** | | | | | **50–59 years** | | | | | **≥60 years** | | | | |
| --- | --- | --- | --- | --- | --- | --- | --- | --- | --- | --- | --- | --- | --- | --- | --- |
|  | **Overall (N=125,255,765)** | **NH White (N=72,017,117)** | **Hispanic (N=24,668,590)** | **NH Black (N=15,753,904)** | **NH Asian (N=8,172,496)** | **Overall (N=42,750,712)** | **NH White (N=29,088,444)** | **Hispanic (N=5,302,128)** | **NH Black (N=4,961,026)** | **NH Asian (N=2,148,302)** | **Overall (N=65,112,014)** | **NH White (N=48,895,667)** | **Hispanic (N=5,456,657)** | **NH Black (N=5,978,889)** | **NH Asian (N=2,858,179)** |
| **≥1 RF^a^** | **17.0 (16.1–18.0)** | **17.7 (16.3–19.2)** | **15.0* (13.5–16.5)** | **19.2 (17.4–21.0)** | **10.0* (8.3–11.7)** | **31.4* (29.2–33.7)** | **29.9 (26.8–32.9)** | **34.1* (30.8–37.3)** | **36.9* (33.9–39.8)** | **24.1* (20.8–27.3)** | **46.9* (45.0–48.8)** | **44.9 (42.5–47.4)** | **50.4* (47.7–53.0)** | **54.0* (51.5–56.6)** | **43.6 (40.0–47.3)** |
| 1 RF | 13.8 (13.0–14.5) | 14.4 (13.2–15.5) | 12.4* (11.0–13.9) | 15.3 (13.7–16.8) | 8.6* (7.0–10.3) | 20.7* (18.7–22.6) | 19.3 (16.8–21.9) | 24.9* (21.7–28.0) | 22.6 (19.8–25.5) | 19.7 (16.8–22.7) | 24.8* (23.2–26.4) | 23.5 (21.6–25.4) | 29.3* (27.1–31.5) | 28.8* (27.0–30.7) | 26.6 (22.7–30.5) |
| 2 RFs | 2.4 (2.0–2.8) | 2.5 (1.9–3.1) | 2.0 (1.5–2.5) | 2.6 (2.0–3.2) | 0.9* (0.5–1.4) | 6.5* (5.5–7.4) | 6.1 (4.8–7.4) | 5.8 (4.1–7.5) | 9.1* (7.2–10.9) | 2.7* (1.7–3.8) | 11.4* (10.4–12.4) | 10.8 (9.6–12.0) | 11.4 (9.9–12.9) | 14.1* (12.2–15.9) | 10.8 (8.5–13.2) |
| 3 RFs | 0.5 (0.4–0.7) | 0.5 (0.3–0.7) | 0.4 (0.2–0.7) | 0.9* (0.6–1.3) | 0.3 (0.1–0.5) | 2.4* (1.9–3.0) | 2.3 (1.6–3.1) | 1.7 (0.9–2.4) | 3.2 (2.0–4.4) | 1.2 (0.3–2.1) | 5.3* (4.7–5.9) | 5.1 (4.4–5.9) | 4.6 (3.5–5.7) | 6.8* (5.7–8.0) | 3.5 (2.0–4.9) |
| ≥4 RFs | 0.3 (0.2–0.4) | 0.4 (0.2–0.6) | 0.1 (0.0–0.3) | 0.4 (0.1–0.7) | 0.1 (0.0–0.3) | 1.9* (1.3–2.5) | 2.1 (1.2–2.9) | 1.7 (0.9–2.5) | 2.0 (1.0–2.9) | 0.4* (0.0–0.9) | 5.4* (4.6–6.2) | 5.5 (4.6–6.5) | 5.0 (3.9–6.0) | 4.3 (3.4–5.2) | 2.7* (1.7–3.8) |
| **Pulmonary and cardiovascular** | **12.0 (11.1–12.8)** | **13.1 (11.8–14.4)** | **8.4* (7.2–9.5)** | **14.4 (12.8–15.9)** | **5.0* (3.9–6.1)** | **19.1* (17.3–20.9)** | **19.3 (16.7–21.9)** | **16.0 (14.0–18.0)** | **22.8 (19.6–20.6)** | **8.8* (6.7–10.8)** | **33.7* (32.0–35.4)** | **34.1 (31.9–36.4)** | **27.8* (25.6–30.1)** | **34.9 (32.7–37.2)** | **21.9* (19.0–24.7)** |
| **Pulmonary** | **10.5 (9.7–11.2)** | **11.6 (10.4–12.8)** | **7.0* (5.9–8.0)** | **12.4 (10.9–13.9)** | **4.1* (3.0–5.2)** | **13.0* (11.4–14.6)** | **13.8 (11.5–16.1)** | **9.5* (7.7–11.2)** | **13.6 (11.4–15.8)** | **5.1* (3.2–7.1)** | **17.2* (15.7–18.6)** | **17.7 (15.8–19.6)** | **12.7* (11.0–14.4)** | **16.8 (15.4–18.2)** | **9.1* (7.1–11.1)** |
| COPD^b^ | 2.5 (2.1–3.0) | 2.9 (2.2–3.6) | 1.1* (0.7–1.5) | 3.0 (1.9–4.2) | 0.7* (0.4–1.1) | 7.1* (5.7–8.4) | 7.9 (6.0–9.7) | 4.1* (2.8–5.4) | 5.6* (4.3–6.9) | 1.1* (0.3–1.9) | 12.1* (10.8–13.4) | 13.1 (11.4–14.7) | 6.8* (5.1–8.4) | 9.6* (8.1–11.1) | 4.5* (3.1–6.0) |
| Asthma  (current)^c^ | 9.0 (8.3–9.6) | 9.8 (8.8–10.9) | 6.3* (5.3–7.3) | 10.5 (9.2–11.9) | 3.8* (2.8–4.7) | 8.8 (7.3–10.2) | 9.1 (7.1–11.1) | 7.5 (5.8–9.1) | 9.9 (8.1–11.7) | 4.5* (2.7–6.3) | 8.6 (7.5–9.7) | 8.2 (6.7–9.6) | 8.6 (7.2–10.1) | 10.9* (9.6–12.2) | 7.4 (5.5–9.3) |
| **Cardiovascular** | **2.2 (1.9–2.5)** | **2.2 (1.7–2.7)** | **1.8 (1.2–2.3)** | **3.0 (2.3–3.6)** | **1.0* (0.5–1.5)** | **8.7* (7.4–9.9)** | **8.1 (6.5–9.7)** | **8.3 (6.6–9.9)** | **13.0* (10.7–15.3)** | **4.2* (2.5–5.8)** | **23.0* (21.7–24.2)** | **23.2 (21.5–24.9)** | **18.7* (16.8–20.6)** | **23.8 (21.6–25.9)** | **15.5* (13.0–18.1)** |
| CHF | 0.6 (0.5–0.7) | 0.4 (0.3–0.6) | 0.6 (0.3–0.9) | 1.4* (0.9–1.8) | 0.3 (0.1–0.6) | 2.1* (1.5–2.6) | 1.8 (1.1–2.6) | 1.8 (0.8–2.9) | 4.2* (3.0–5.5) | 0.3* (0.0–0.7) | 6.9* (6.2–7.6) | 6.8 (5.9–7.8) | 5.7 (4.6–6.8) | 8.7* (7.1–10.3) | 3.5* (2.3–4.7) |
| CHD | 0.5 (0.3–0.7) | 0.6 (0.3–0.9) | 0.4 (0.1–0.6) | 0.5 (0.2–0.7) | 0.3 (0.0–0.5) | 2.9* (2.1–3.7) | 3.1 (2.0–4.2) | 2.1 (1.1–3.2) | 2.3 (1.5–3.1) | 1.7 (0.5–2.8) | 10.3* (9.1–11.6) | 11.2  (9.6–12.9) | 7.7* (6.1–9.3) | 6.2* (5.2–7.3) | 7.1* (5.3–8.9) |
| Stroke | 0.7 (0.6–0.9) | 0.8 (0.5–1.0) | 0.6 (0.3–1.0) | 1.0 (0.6–1.4) | 0.2* (0.0–0.5) | 3.4* (2.7–4.2) | 2.8 (1.8–3.9) | 4.0 (2.8–5.2) | 6.5* (4.7–8.3) | 1.0* (0.2–1.7) | 7.7* (7.0–8.4) | 7.3 (6.4–8.2) | 6.0 (4.9–7.1) | 10.6* (9.2–12.0) | 5.4 (3.8–7.0) |
| Angina pectoris | 0.5 (0.4–0.7) | 0.7 (0.4–0.9) | 0.3 (0.1–0.5) | 0.5 (0.2–0.8) | 0.3* (0.0–0.5) | 2.4* (1.7–3.1) | 2.8 (1.8–3.8) | 1.2* (0.5–1.8) | 2.2 (1.2–3.1) | 1.1* (0.3–1.9) | 5.3* (4.7–5.8) | 5.5 (4.9–6.2) | 5.1 (3.9–6.3) | 3.1* (2.4–3.9) | 3.6 (1.9–5.3) |
| MI | 0.6 (0.4–0.7) | 0.6 (0.3–0.8) | 0.4 (0.2–0.7) | 0.8 (0.5–1.2) | 0.3 (0.0–0.6) | 3.8* (2.9–4.7) | 4.0 (2.8–5.2) | 2.4* (1.4–3.4) | 3.9 (2.7–5.0) | 2.2 (0.9–3.5) | 8.8* (8.0–9.6) | 8.9 (7.8–10.0) | 8.0 (6.4–9.6) | 7.4 (6.3–8.5) | 4.0* (2.7–5.3) |
| **Endocrine and metabolic** | **6.7 (6.2–7.2)** | **6.3 (5.5–7.1)** | **7.9* (6.7–9.0)** | **6.8 (5.7–7.8)** | **5.9 (4.7–7.1)** | **18.2* (16.4–20.0)** | **16.4 (14.1–18.6)** | **23.5* (20.7–26.3)** | **21.4* (19.1–23.6)** | **17.8 (14.8–20.9)** | **25.8* (24.3–27.2)** | **22.5 (20.9–24.2)** | **35.3* (32.7–37.9)** | **36.1* (33.7–38.4)** | **31.2* (27.3–35.1)** |
| Diabetes^d^ | 4.0 (3.7–4.4) | 3.4 (2.8–4.1) | 5.3* (4.3–6.2) | 5.3* (4.5–6.2) | 3.5 (2.5–4.4) | 13.6* (11.9–15.3) | 11.5 (9.2–13.8) | 18.8* (16.1–21.4) | 18.1* (15.9–20.3) | 14.7 (11.8–17.7) | 21.2* (20.1–22.4) | 18.2 (17.0–19.5) | 30.3* (27.7–32.9) | 30.6* (28.5–32.7) | 27.1* (23.5–30.8) |
| Renal disease | 1.7 (1.4–2.1) | 1.8 (1.3–2.3) | 2.0 (1.5–2.4) | 1.5 (1.0–2.0) | 1.1 (0.5–1.7) | 2.9* (2.0–3.8) | 2.6 (1.4–3.9) | 2.3 (1.2–3.5) | 5.2* (3.5–6.9) | 2.0 (0.7–3.3) | 5.6* (4.9–6.3) | 5.1 (4.3–5.9) | 6.5 (5.2–7.8) | 8.5* (7.1–9.9) | 4.2 (2.5–6.0) |
| Liver disease  (current)^e^ | 1.5 (1.2–1.7) | 1.6 (1.2–2.0) | 1.4 (1.0–1.9) | 0.4* (0.2–0.6) | 1.6 (1.0–2.2) | 3.7* (3.0–4.4) | 3.8 (2.9–4.8) | 5.2 (4.0–6.4) | 1.4* (0.6–2.2) | 2.2 (0.9–3.4) | 2.7* (2.2–3.2) | 2.4 (1.8–3.0) | 4.2* (3.1–5.3) | 2.6 (1.9–3.4) | 4.2* (2.9–5.6) |

Notes: Diagnosed estimates include individuals who reported having a diagnosis of a RF in the interview component. Hispanic group defined in NHANES as Mexican American or other Hispanic. Other race and ethnicity/multi-racial results not presented.

^a^Summary measures of RFs include those measurable via NHANES: COPD, asthma (current), CHF, CHD, stroke, angina pectoris, MI, diabetes, renal disease, and/or liver disease (current).

^b^Defined as a “yes” response to any of the following questionnaire items: MCQ160o (Has a doctor or other health professional ever told [you/SP] that [you/s/he] had COPD? 2013–2014 and 2015–2016 only) or MCQ170k (Do you still have chronic bronchitis? 2011–2012, 2013–2014, and 2015–2016 only), MCQ160g (Has a doctor or other health professional ever told [you/SP] that [you/s/he] had emphysema? 2011–2012, 2013–2014, and 2015–2016 only), or MCQ160p ([Have you/Has SP] ever been told by a doctor or other health professional that [you/he/she] had chronic obstructive pulmonary disease or COPD, emphysema, or chronic bronchitis? 2017–March 2020 only).

^c^Only asked among respondents who answered “yes” to having ever received a diagnosis of asthma. Respondents who did not report ever receiving a diagnosis of asthma are included in the “no” category.

^d^Respondents who reported having borderline diabetes are included in the “no” category.

^e^Only asked among respondents who answered “yes” to having ever received a diagnosis of liver disease. Respondents who did not report ever receiving a diagnosis of liver disease are included in the “no” category.

*Statistically significant (p<0.05) based on pairwise chi-square analysis on 2x2 tables comparing proportion of respondents with each RF in respective group to the reference group. For the overall results, age 20–49 years is the reference group. Within each age group, non-Hispanic White is the reference group.

Abbreviations: CHD, coronary heart disease; CHF, congestive heart failure; CI, confidence interval; COPD, chronic obstructive pulmonary disease; MI, myocardial infarction; NH, non-Hispanic; NHANES, National Health and Nutrition Examination Survey; RF, risk factor; RSV, respiratory syncytial virus; SP, sample person; US, United States.

### Supplementary Table 3. Percentage of adults aged ≥20 years with diagnosed RFs for severe RSV disease, by PIR and age group

| RFs, % (95% CI) | **20–49 years** | | | | | | **50–59 years** | | | | | | **≥60 years** | | | | | |
| --- | --- | --- | --- | --- | --- | --- | --- | --- | --- | --- | --- | --- | --- | --- | --- | --- | --- | --- |
|  | **≥5 (N=26,558,464)** | **4–<5 (N=11,947,071)** | **3–<4 (N=14,450,782)** | **2–<3 (N=17,476,537)** | **1–<2 (N=23,824,171)** | **<1 (N=20,197,294)** | **≥5 (N=14,143,572)** | **4–<5 (N=3,827,218)** | **3–<4 (N=4,552,411)** | **2–<3 (N=4,988,568)** | **1–<2 (N=6,299,654)** | **<1 (N=5,066,789)** | **≥5 (N=16,003,006)** | **4–<5 (N=5,875,108)** | **3–<4 (N=7,715,458)** | **2–<3 (N=9,626,945)** | **1–<2 (N=13,010,079)** | **<1 (N=6,113,482)** |
| **≥1 RF^a^** | **13.2 (11.3–15.1)** | **15.3 (12.3–18.4)** | **14.8 (12.2–17.4)** | **17.3* (15.0–19.6)** | **18.6* (16.5–20.8)** | **22.7* (20.5–24.9)** | **19.4 (15.7–23.1)** | **23.4 (16.9–30.0)** | **32.3* (25.4–39.1)** | **37.9* (30.3–45.4)** | **39.1* (35.3–42.9)** | **49.1* (44.0–54.2)** | **35.4 (31.4–39.4)** | **41.0 (34.8–47.2)** | **44.2* (38.6–49.8)** | **49.5* (45.1–53.9)** | **57.6* (54.7–60.5)** | **58.1* (54.2–61.9)** |
| 1 RF | 12.0 (10.2–13.8) | 12.6 (9.9–15.3) | 12.4 (10.0–14.8) | 14.0 (11.9–16.1) | 14.3 (12.7–15.9) | 16.9* (15.0–18.8) | 15.1 (11.8–18.4) | 13.3 (8.5–18.2) | 23.3* (17.5–29.2) | 25.0* (18.1–32.0) | 24.6* (21.5–27.7) | 25.9* (22.0–29.9) | 20.4 (17.1–23.6) | 22.9 (17.7–28.2) | 24.9 (20.8–29.1) | 25.6* (22.0–29.1) | 28.3* (25.8–30.9) | 27.2* (23.7–30.8) |
| 2 RFs | 1.1 (0.5–1.6) | 2.6* (1.2–4.0) | 1.9 (1.0–2.8) | 2.3* (1.2–3.4) | 3.3* (2.3–4.3) | 3.7* (2.9–4.5) | 3.2 (2.0–4.5) | 6.8* (4.1–9.5) | 5.9 (2.5–9.3) | 8.2* (4.1–12.2) | 8.6* (6.1–11.0) | 11.8* (8.6–15.0) | 9.3 (7.2–11.4) | 8.4 (4.4–12.3) | 10.6 (7.6–13.6) | 11.9 (9.5–14.4) | 13.3* (11.4–15.3) | 15.2* (12.4–18.0) |
| 3 RFs | 0.1 (0.0–0.2) | 0.2 (0.0–0.4) | 0.4* (0.0–0.7) | 0.4* (0.1–0.7) | 0.5* (0.3–0.7) | 1.6* (1.0–2.1) | 0.4 (0.0–0.9) | 0.8 (0.0–1.8) | 2.2* (0.0–4.4) | 3.5* (1.1–5.9) | 3.5* (2.1–4.8) | 5.4* (3.7–7.2) | 3.1 (2.1–4.2) | 5.4 (2.7–8.2) | 4.3 (2.5–6.0) | 5.3* (3.5–7.1) | 7.6* (6.3–9.0) | 6.0* (4.5–7.5) |
| ≥4 RFs | 0.0 (0.0–0.1) | 0.0 (0.0–0.0) | 0.1 (0.0–0.4) | 0.6* (0.0–1.2) | 0.6* (0.3–0.9) | 0.5* (0.2–0.8) | 0.7 (0.0–1.4) | 2.5 (0.0–5.3) | 0.8 (0.1–1.4) | 1.1 (0.0–2.6) | 2.4* (1.6–3.3) | 6.0* (3.4–8.6) | 2.6 (0.9–4.3) | 4.2 (2.1–6.4) | 4.4 (2.8–5.9) | 6.7* (4.4–9.1) | 8.3* (6.6–10.0) | 9.6* (7.7–11.6) |
| **Pulmonary and cardiovascular** | **8.3 (6.7–9.9)** | **11.4* (8.9–13.8)** | **9.7 (7.5–12.0)** | **12.5* (10.3–14.7)** | **13.6* (11.6–15.5)** | **16.7* (15.1–18.3)** | **11.6 (8.1–15.0)** | **12.2 (7.7–16.6)** | **18.9* (13.8–24.0)** | **20.5* (14.5–26.6)** | **25.9* (21.9–29.9)** | **33.0* (27.3–38.6)** | **24.5 (20.8–28.2)** | **28.5 (23.1–33.9)** | **31.0 (25.7–36.3)** | **37.7* (33.3–42.2)** | **42.3* (39.3–45.3)** | **41.6* (37.4–45.9)** |
| **Pulmonary** | **7.4 (5.9–8.9)** | **10.7* (8.3–13.1)** | **8.4 (6.2–10.5)** | **10.9* (8.9–12.9)** | **11.5* (9.6–13.5)** | **14.4* (12.8–15.9)** | **8.6 (5.6–11.5)** | **6.9 (3.3–10.6)** | **12.8 (7.6–18.0)** | **12.1 (7.6–16.6)** | **17.3* (14.4–20.1)** | **22.8* (17.8–27.8)** | **11.2 (8.1–14.2)** | **14.9 (10.7–19.0)** | **17.0* (13.2–20.8)** | **18.2* (15.1–21.4)** | **22.7* (20.4–25.0)** | **23.9* (19.6–28.2)** |
| COPD^b^ | 0.8 (0.3–1.3) | 1.7 (0.6–2.9) | 1.4 (0.4–2.3) | 1.8* (0.9–2.7) | 3.7* (2.5–4.8) | 5.1* (4.1–6.2) | 2.0 (1.0–2.9) | 2.9 (0.4–5.4) | 6.1* (2.0–10.1) | 8.4* (3.8–12.9) | 11.6* (9.0–14.1) | 16.3* (10.9–21.8) | 7.1 (4.8–9.4) | 9.5 (6.3–12.7) | 11.9* (8.8–15.1) | 13.2* (10.3–16.1) | 17.7* (15.2–20.2) | 17.1* (13.1–21.1) |
| Asthma  (current)^c^ | 6.8 (5.4–8.3) | 9.9* (7.6–12.2) | 7.8 (5.7–9.9) | 10.0* (8.0–11.9) | 9.4* (7.8–11.0) | 11.2* (9.8–12.5) | 7.4 (4.4–10.4) | 4.4 (1.5–7.3) | 8.8 (4.5–13.0) | 7.5 (4.0–11.0) | 9.3 (7.1–11.5) | 14.9* (11.8–18.0) | 6.1 (3.6–8.6) | 8.7 (5.0–12.3) | 6.8 (4.0–9.6) | 8.5 (6.2–10.9) | 10.7* (9.0–12.4) | 13.5* (10.8–16.3) |
| **Cardiovascular** | **0.9 (0.5–1.4)** | **0.7 (0.2–1.3)** | **1.6 (0.8–2.3)** | **2.2* (1.3–3.1)** | **3.3* (2.5–4.1)** | **3.6* (2.8–4.4)** | **3.5 (1.8–5.2)** | **6.3 (2.9–9.7)** | **8.1* (4.8–11.4)** | **10.4* (5.2–15.6)** | **13.4* (10.0–16.7)** | **18.4* (14.2–22.5)** | **16.7 (13.2–20.1)** | **18.2 (14.4–22.0)** | **21.4 (17.2–25.5)** | **27.1* (23.3–30.9)** | **28.8* (26.1–31.5)** | **27.8* (25.0–30.7)** |
| CHF | 0.1 (0.0–0.2) | 0.5* (0.0–1.0) | 0.5* (0.1–0.9) | 0.1 (0.0–0.2) | 1.1* (0.6–1.6) | 1.1* (0.7–1.5) | 0.6 (0.0–1.3) | 0.8 (0.0–2.2) | 1.3 (0.0–2.7) | 2.3 (0.4–4.2) | 2.8* (1.8–3.8) | 5.9* (3.7–8.1) | 3.0 (2.0–4.1) | 5.4* (2.6–8.1) | 6.5* (4.5–8.5) | 8.2* (5.7–10.7) | 10.8* (9.3–12.3) | 10.4* (8.4–12.5) |
| CHD | 0.2 (0.0–0.4) | 0.1 (0.0–0.2) | 0.4 (0.0–0.8) | 0.8 (0.1–1.4) | 0.5 (0.3–0.8) | 0.6* (0.4–0.9) | 1.9 (0.8–3.0) | 3.3 (0.7–5.9) | 0.8 (0.0–1.6) | 3.3 (0.0–7.1) | 3.9 (1.9–5.9) | 5.3* (2.6–8.0) | 9.1 (6.3–12.0) | 7.4 (4.5–10.3) | 12.1 (8.7–15.5) | 10.7 (7.9–13.4) | 11.9 (9.5–14.3) | 10.4 (8.6–12.2) |
| Stroke | 0.3 (0.1–0.6) | 0.1 (0.0–0.2) | 0.6 (0.1–1.2) | 0.6 (0.2–1.0) | 1.1* (0.7–1.5) | 1.5* (0.9–2.2) | 1.5 (0.3–2.7) | 0.8 (0.10–1.5) | 3.8 (1.2–6.4) | 2.8 (1.0–4.5) | 4.9* (3.5–6.3) | 9.2* (6.0–12.4) | 4.6 (3.3–5.9) | 6.5 (4.2–8.7) | 5.7 (3.9–7.5) | 9.3* (7.1–11.5) | 10.8* (9.3–12.4) | 10.7* (8.2–13.2) |
| Angina  pectoris | 0.2 (0.0–0.4) | 0.1 (0.0–0.2) | 0.4 (0.0–0.8) | 1.2* (0.4–1.9) | 0.7* (0.4–1.1) | 0.8* (0.4–1.3) | 0.7 (0.0–1.5) | 3.5* (0.5–6.5) | 2.5 (0.2–4.8) | 2.6 (0.4–4.7) | 3.5* (1.5–5.5) | 4.4* (2.4–6.5) | 3.7 (2.3–5.2) | 4.5 (2.6–6.5) | 3.6 (2.0–5.2) | 6.4* (4.3–8.4) | 6.5* (5.0–8.0) | 7.3* (5.3–9.4) |
| MI | 0.1 (0.0–0.3) | 0.1 (0.0–0.3) | 0.1 (0.0–0.3) | 0.6* (0.1–1.1) | 0.8* (0.5–1.1) | 1.1* (0.7–1.6) | 1.7 (0.4–3.0) | 4.4 (1.2–7.6) | 2.8 (1.0–4.7) | 2.6 (0.7–4.5) | 6.3* (3.7–8.9) | 8.3* (5.6–10.9) | 6.7 (4.5–8.9) | 5.9 (3.4–8.4) | 7.5 (4.9–10.0) | 9.2 (6.6–11.9) | 12.3* (10.4–14.2) | 10.4* (8.5–12.2) |
| **Endocrine and metabolic** | **5.7 (4.5–7.0)** | **5.3 (3.4–7.3)** | **5.9 (4.4–7.5)** | **6.5 (5.0–8.0)** | **7.0 (5.7–8.3)** | **8.9* (7.6–10.2)** | **10.3 (7.7–12.9)** | **17.6* (11.2–24.0)** | **18.5* (13.2–23.8)** | **25.5* (18.5–32.5)** | **20.1* (17.2–23.0)** | **27.9* (22.8–33.0)** | **18.7 (15.6–21.8)** | **23.5 (18.3–28.7)** | **22.1 (18.2–26.0)** | **25.7* (22.0–29.4)** | **32.0* (29.3–34.6)** | **36.2* (32.5–39.8)** |
| Diabetes^d^ | 3.7 (2.6–4.8) | 3.0 (1.5–4.5) | 4.4 (3.1–5.7) | 3.7 (2.7–4.8) | 3.9 (3.1–4.7) | 5.3 (4.3–6.2) | 7.4 (5.1–9.7) | 14.0* (9.1–18.9) | 15.8* (10.5–21.2) | 20.9* (14.4–27.4) | 13.4* (11.6–15.2) | 20.7* (16.3–25.2) | 15.5 (12.5–18.5) | 20.9* (16.0–25.8) | 17.2 (13.6–20.8) | 22.5* (19.0–26.0) | 25.7* (23.4–28.1) | 29.4* (26.2–32.7) |
| Renal disease | 1.0 (0.5–1.5) | 1.4 (0.4–2.4) | 1.2 (0.4–2.0) | 1.8 (0.9–2.7) | 2.3* (1.5–3.1) | 2.2* (1.3–3.2) | 2.1 (0.7–3.5) | 2.6 (0.0–5.4) | 1.8 (0.6–3.1) | 2.9 (0.8–5.0) | 4.1 (2.3–5.9) | 3.3 (1.9–4.8) | 3.0 (1.7–4.4) | 3.9 (1.9–5.9) | 5.9* (3.8–8.0) | 5.5* (3.8–7.3) | 7.8* (6.6–9.0) | 8.6* (6.0–11.1) |
| Liver disease  (current)^e^ | 1.2 (0.4–1.9) | 1.3 (0.3–2.4) | 0.9 (0.2–1.5) | 1.7 (0.9–2.6) | 1.5 (1.0–2.0) | 2.4 (1.7–3.1) | 1.4 (0.3–2.4) | 3.2 (0.3–6.2) | 1.6 (0.0–3.1) | 4.8* (1.2–8.5) | 4.7* (2.5–6.9) | 7.9* (3.6–12.2) | 1.7 (0.7–2.7) | 1.9 (0.8–3.0) | 3.1 (1.6–4.6) | 2.3 (1.2–3.3) | 3.4* (2.3–4.5) | 4.8* (2.0–7.5) |

Notes: Diagnosed estimates include individuals who reported having a diagnosis of a RF in the interview component. PIR calculated by NHANES by dividing family (or individual) income by the US Health and Human Services poverty guidelines relevant to the survey year; a ratio <1 represents family income below poverty level. Missing PIR results not presented.

^a^Summary measures of RFs include those measurable via NHANES: COPD, asthma (current), CHF, CHD, stroke, angina pectoris, MI, diabetes, renal disease, and/or liver disease (current).

^b^Defined as a “yes” response to any of the following questionnaire items: MCQ160o (Has a doctor or other health professional ever told [you/SP] that [you/s/he] had COPD? 2013–2014 and 2015–2016 only) or MCQ170k (Do you still have chronic bronchitis? 2011–2012, 2013–2014, and 2015–2016 only), MCQ160g (Has a doctor or other health professional ever told [you/SP] that [you/s/he] had emphysema? 2011–2012, 2013–2014, and 2015–2016 only), or MCQ160p ([Have you/Has SP] ever been told by a doctor or other health professional that [you/he/she] had chronic obstructive pulmonary disease or COPD, emphysema, or chronic bronchitis? 2017–March 2020 only).

^c^Only asked among respondents who answered “yes” to having ever received a diagnosis of asthma. Respondents who did not report ever receiving a diagnosis of asthma are included in the “no” category.

^d^Respondents who reported having borderline diabetes are included in the “no” category.

^e^Only asked among respondents who answered “yes” to having ever received a diagnosis of liver disease. Respondents who did not report ever receiving a diagnosis of liver disease are included in the “no” category.

*Statistically significant (p<0.05) based on pairwise chi-square analysis on 2x2 tables comparing proportion of respondents with each RF in respective group to the reference group (i.e., PIR ≥5).

Abbreviations: CHD, coronary heart disease; CHF, congestive heart failure; CI, confidence interval; COPD, chronic obstructive pulmonary disease; MI, myocardial infarction; NHANES, National Health and Nutrition Examination Survey; PIR, poverty income ratio; RF, risk factor; RSV, respiratory syncytial virus; SP, sample person; US, United States.

### Supplementary Table 4. Percentage of adults aged ≥20 years with undiagnosed diabetes and renal disease

| RF, % (95% CI) | **Overall and by race and ethnicity** | | | | | | | | | | | | | | | | | | | | | | | | | | | | | | | |
| --- | --- | --- | --- | --- | --- | --- | --- | --- | --- | --- | --- | --- | --- | --- | --- | --- | --- | --- | --- | --- | --- | --- | --- | --- | --- | --- | --- | --- | --- | --- | --- | --- |
|  | **20–49 years** | | | | | | | | | | | **50–59 years** | | | | | | | | | | | **≥60 years** | | | | | | | | | |
|  | **Overall**  **(N=125,255,765)** | | **NH White**  **(N=72,017,117)** | | **Hispanic**  **(N=24,668,590)** | | **NH Black**  **(N=15,753,904)** | | **NH Asian**  **(N=8,172,496)** | | **Overall**  **(N=42,750,712)** | | | **NH White**  **(N=29,088,444)** | | **Hispanic**  **(N=5,302,128)** | | **NH Black**  **(N=4,961,026)** | | **NH Asian**  **(N=2,148,302)** | | | **Overall**  **(N=65,112,014)** | | **NH White**  **(N=48,895,667)** | | **Hispanic**  **(N=5,456,657)** | | **NH Black**  **(N=5,978,889)** | | **NH Asian**  **(N=2,858,179)** | |
| Diabetes^a^ | 2.1 (1.8–2.4) | | 1.4 (1.0–1.8) | | 3.2* (2.4–3.9) | | 3.1* (2.2–3.9) | | 2.9* (2.0–3.9) | | 4.7* (3.6–5.7) | | | 3.5 (2.2–4.8) | | 8.5* (6.3–10.7) | | 7.0* (5.1–8.9) | | 7.1* (4.6–9.6) | | | 6.6* (5.8–7.4) | | 5.6 (4.6–6.6) | | 12.1* (9.9–14.2) | | 9.9* (8.3–11.5) | | 11.6* (8.4–14.8) | |
| Renal disease^b^ | 6.4 (5.9–6.9) | | 5.4 (4.7–6.2) | | 8.1* (7.0–9.3) | | 8.3* (7.2–9.5) | | 6.2 (4.8–7.7) | | 11.1* (9.5–12.7) | | | 9.4 (7.3–11.6) | | 11.3 (8.8–13.7) | | 18.3* (15.4–21.2) | | 11.6 (9.0–14.3) | | | 25.6* (24.2–27.0) | | 24.5 (22.9–26.2) | | 24.2 (21.7–26.7) | | 37.3* (34.5–40.1) | | 23.5 (19.8–27.3) | |
| RF, % (95% CI) | **By PIR** | | | | | | | | | | | | | | | | | | | | | | | | | | | | | | | |
|  | **20–49 years** | | | | | | | | | | | **50–59 years** | | | | | | | | | | **≥60 years** | | | | | | | | | | |
|  | **≥5**  **(N=26,558,464)** | **4–<5**  **(N=11,947,071)** | | **3–<4**  **(N=14,450,782)** | | **2–<3**  **(N=17,476,537)** | | **1–<2**  **(N=23,824,171)** | | **<1**  **(N=20,197,294)** | | **≥5**  **(N=14,143,572)** | **4–<5**  **(N=3,827,218)** | | **3–<4**  **(N=4,552,411)** | | **2–<3**  **(N=4,988,568)** | | **1–<2**  **(N=6,299,654)** | | **<1**  **(N=5,066,789)** | **≥5**  **(N=16,003,006)** | | **4–<5**  **(N=5,875,108)** | | **3–<4**  **(N=7,715,458)** | | **2–<3**  **(N=9,626,945)** | | **1–<2**  **(N=13,010,079)** | | **<1**  **(N=6,113,482)** |
| Diabetes^a^ | 1.0 (0.5–1.4) | 2.2* (1.1–3.4) | | 2.4* (1.3–3.5) | | 1.6 (0.9–2.2) | | 3.3* (2.4–4.2) | | 2.8* (2.0–3.5) | | 3.6 (1.8–5.3) | 4.0 (1.0–6.9) | | 4.8 (1.7–7.8) | | 4.4 (1.6–7.1) | | 6.1 (3.6–8.5) | | 8.5* (5.3–11.6) | 3.6 (2.1–5.1) | | 6.1 (3.5–8.6) | | 7.6* (5.1–10.2) | | 7.0* (4.7–9.2) | | 7.9* (6.2–9.6) | | 11.4* (8.6–14.3) |
| Renal disease^b^ | 4.9 (3.7–6.1) | 4.1 (2.5–5.7) | | 5.2 (3.3–7.1) | | 6.3 (4.9–7.8) | | 7.8* (6.7–8.8) | | 8.8* (7.8–9.9) | | 6.7 (4.6–8.8) | 10.9 (5.4–16.3) | | 8.4 (4.7–12.2) | | 14.2* (8.4–20.0) | | 14.5* (11.2–17.9) | | 19.8* (16.1–23.5) | 19.9 (16.7–23.1) | | 19.9 (14.9–24.9) | | 21.3 (17.3–25.3) | | 28.5* (25.4–31.6) | | 32.9* (30.2–35.5) | | 35.3* (31.8–38.9) |

Notes: Undiagnosed estimates include individuals who reported not having a diagnosis of a RF in the interview component, but the corresponding examination/laboratory component indicates presence of condition. Hispanic group defined in NHANES as Mexican American or other Hispanic. PIR calculated by NHANES by dividing family (or individual) income by the US Health and Human Services poverty guidelines relevant to the survey year; a ratio <1 represents family income below poverty level. Other race and ethnicity/multi-racial results and missing PIR results not presented.

^a^Undiagnosed diabetes defined as no self-reported diagnosis in the interview component and plasma fasting glucose >126 mg/dL (≥7.0 mmol/L), or HbA1C >6.5%, or a two-hour oral glucose tolerance test result ≥200 mg/dL. The two-hour oral glucose tolerance test is not available in 2017–March 2020.

^b^Undiagnosed renal disease defined as no self-reported diagnosis in the interview component and eGFR <60 mg/dL (CKD-EPI formula) or urine albumin creatinine ratio ≥30 mg/g.

*Statistically significant (p<0.05) based on pairwise chi-square analysis on 2x2 tables comparing proportion of respondents with each RF in respective group to the reference group. For the overall results, age 20–49 years is the reference group. Within each age group, non-Hispanic White and PIR ≥5 are the reference groups.

Abbreviations: CI, confidence interval; CKD-EPI, Chronic Kidney Disease Epidemiology Collaboration; eGFR, estimated glomerular filtration rates; HbA1c, hemoglobin A1c; NH, non-Hispanic; NHANES, National Health and Nutrition Examination Survey; PIR, poverty income ratio; RF, risk factor; US, United States.

### Supplementary Table 5. Combined percentage of adults aged ≥20 years with ≥1 diagnosed RF for severe RSV disease or undiagnosed diabetes or renal disease^a^

|  | **20–49 years (N=125,255,765)** | **50–59 years (N=42,750,712)** | **≥60 years (N=65,095,825)** |
| --- | --- | --- | --- |
| RFs, % (95% CI) | **Overall** | | |
| **≥1 RF** | **22.4 (21.4–23.5)** | **38.1* (35.7–40.6)** | **58.4* (56.6–60.2)** |
| 1 RF | 17.5 (16.6–18.3) | 22.7* (20.7–24.6) | 28.5* (26.9–30.1) |
| 2 RFs | 3.7 (3.3–4.2) | 9.7* (8.4–11.0) | 15.5* (14.4–16.6) |
| 3 RFs | 0.8 (0.6–1.0) | 3.3* (2.5–4.1) | 6.7* (6.0–7.5) |
| ≥4 RFs | 0.4 (0.3–0.6) | 2.4* (1.8–3.1) | 7.7* (6.8–8.6) |
| ≥1 RF, % (95% CI) | **By race and ethnicity** | | |
| Non-Hispanic White | 22.2 (20.6–23.8) | 35.2 (31.8–38.5) | 56.4 (54.1–58.6) |
| Hispanic | 22.1 (20.5–23.7) | 42.2* (39.1–45.2) | 61.2* (58.5–63.9) |
| Non-Hispanic Black | 25.8* (23.8–27.9) | 47.7* (44.5–50.8) | 68.4* (66.0–70.8) |
| Non-Hispanic Asian | 16.3* (14.3–18.3) | 33.9 (30.3–37.5) | 56.6 (52.7–60.6) |
| ≥1 RF, % (95% CI) | **By PIR** | | |
| ≥5 | 17.4 (15.3–19.5) | 24.8 (20.9–28.8) | 46.0 (41.9–50.1) |
| 4–<5 | 19.8 (16.7–22.8) | 29.0 (22.7–35.4) | 52.3 (46.1–58.5) |
| 3–<4 | 19.6 (16.6–22.7) | 36.5* (29.9–43.0) | 56.0* (50.3–61.7) |
| 2–<3 | 22.6* (20.2–24.9) | 45.9* (39.0–52.8) | 63.4* (59.2–67.6) |
| 1–<2 | 25.3* (23.3–27.3) | 48.5* (44.3–52.8) | 68.9* (66.6–71.3) |
| <1 | 29.1* (26.9–31.3) | 59.1* (54.6–63.7) | 71.2* (67.7–74.6) |

Notes: Hispanic group defined in NHANES as Mexican American or other Hispanic. PIR calculated by NHANES by dividing family (or individual) income by the US Health and Human Services poverty guidelines relevant to the survey year; a ratio <1 represents family income below poverty level. Other race and ethnicity/multi-racial results and missing PIR results not presented.

^a^Diagnosed estimates include individuals who reported having a diagnosis of a RF in the interview component. Undiagnosed estimates include individuals who reported not having a diagnosis of a RF in the interview component, but the corresponding examination/laboratory component indicates presence of condition. Overall estimates include individuals who reported having received a diagnosis of a given RF in the interview component, individuals who reported not having a diagnosis of a RF in the interview component but the corresponding examination/laboratory component indicates presence of condition, and individuals who did not answer or responded “don't know” to the interview component but the corresponding examination/laboratory component indicates presence of a given RF. Of the 10 included RFs for severe RSV disease measurable via NHANES (COPD, asthma [current], CHF, CHD, stroke, angina pectoris, MI, diabetes, renal disease, and/or liver disease [current]), examination and laboratory tests were only available to determine the presence of diabetes and renal disease.

*Statistically significant (p<0.05) based on pairwise chi-square analysis on 2x2 tables comparing proportion of respondents with each RF in respective group to the reference group. For the overall results, age 20–49 years is the reference group. For results by race and ethnicity and by PIR, non-Hispanic White and PIR ≥5 are the reference groups (within each age group).

Abbreviations: CHD, coronary heart disease; CHF, congestive heart failure; CI, confidence interval; COPD, chronic obstructive pulmonary disease; NHANES, National Health and Nutrition Examination Survey; MI, myocardial infarction; PIR, poverty income ratio; RF, risk factor; RSV, respiratory syncytial virus; US, United States.

### Supplementary Table 6. Mean age at identification of undiagnosed diabetes and renal disease, among adults aged ≥20 years with ≥1 undiagnosed RF for severe RSV disease

| Mean age at RF identification, years (95% CI) | **Overall and by race and ethnicity** | | | | | | | | | |
| --- | --- | --- | --- | --- | --- | --- | --- | --- | --- | --- |
|  | **Overall**  **(N=37,396,840)** | | **Non-Hispanic White**  **(N=23,813,186)** | | **Hispanic**  **(N=5,118,292)** | | **Non-Hispanic Black**  **(N=5,192,306)** | | **Non-Hispanic Asian**  **(N=1,853,604)** | |
| Diabetes^a^ | 56.5 (55.2–57.7) | | 60.2 (58.2–62.1) | | 51.7* (49.8–53.6) | | 53.3* (51.2–55.3) | | 54.9* (51.7–58.0) | |
| Renal disease^b^ | 59.2 (58.4–60.0) | | 62.1 (61.0–63.2) | | 50.2* (48.6–51.8) | | 56.7* (55.6–57.8) | | 55.7* (52.8–58.6) | |
| Mean age at RF identification, years (95% CI) | **By PIR** | | | | | | | | | |
|  | **≥5**  **(N=7,659,532)** | **4–<5**  **(N=3,163,279)** | | **3–<4**  **(N=3,950,246)** | | **2–<3**  **(n=5,656,817)** | | **1–<2**  **(N=8,260,822)** | | **<1**  **(N=5,683,339)** |
| Diabetes^a^ | 57.7 (55.2–60.2) | 55.8 (51.4–60.2) | | 56.7 (53.1–60.3) | | 60.1 (56.8–63.5) | | 54.7 (52.0–57.3) | | 53.6 (50.7–56.4) |
| Renal disease^b^ | 59.8 (57.9–61.8) | 60.8 (58.1–63.4) | | 59.8 (56.8–62.9) | | 60.6 (58.4–62.7) | | 60.4 (58.9–61.8) | | 54.1* (52.2–55.9) |

Notes: Age at identification of undiagnosed estimates include age at screening among individuals who reported not having a diagnosis of a RF in the interview component, but the corresponding examination/laboratory component indicates presence of condition. Hispanic group defined in NHANES as Mexican American or other Hispanic. PIR calculated by NHANES by dividing family (or individual) income by the US Health and Human Services poverty guidelines relevant to the survey year; a ratio <1 represents family income below poverty level. Other race and ethnicity/multi-racial results and missing PIR results not presented.

^a^Undiagnosed diabetes defined as no self-reported diagnosis in the interview component and plasma fasting glucose >126 mg/dL (≥7.0 mmol/L), or HbA1C >6.5%, or a two-hour oral glucose tolerance test result ≥200 mg/dL. The two-hour oral glucose tolerance test is not available in 2017–March 2020.

^b^Undiagnosed renal disease defined as no self-reported diagnosis in the interview component and eGFR <60 mg/dL (CKD-EPI formula) or urine albumin creatinine ratio ≥30 mg/g.

*Statistically significant (p<0.05) based on Wald-pairwise t-test analysis on 2x2 tables comparing mean age at diagnosis in respective group to the reference group (i.e., non-Hispanic White or PIR ≥5).

Abbreviations: CI, confidence interval; CKD-EPI, Chronic Kidney Disease Epidemiology Collaboration; eGFR, estimated glomerular filtration rates; HbA1c, hemoglobin A1c; NHANES, National Health and Nutrition Examination Survey; PIR, poverty income ratio; RF, risk factor; US, United States.

### Supplementary Table 7. Percentage of adults aged ≥20 years with ≥1 RF for severe RSV disease diagnosed before age 50 years, stratified by individual RF

| RFs, % (95% CI) | **Overall and by race and ethnicity** | | | | | | | | | |
| --- | --- | --- | --- | --- | --- | --- | --- | --- | --- | --- |
|  | **Overall**  **(N=58,999,617)** | | **Non-Hispanic White**  **(N=38,994,125)** | | **Hispanic (N=7,489,193)** | | **Non-Hispanic Black**  **(N=7,420,066)** | | **Non-Hispanic Asian**  **(N=2,386,308)** | |
| **≥1 RF^a^** | **60.0 (58.0–62.0)** | | **56.7 (53.9–59.4)** | | **71.1* (68.3–74.0)** | | **66.9* (64.3–69.4)** | | **57.7 (53.1–62.2)** | |
| 1 RF | 63.1 (60.8–65.5) | | 60.3 (56.9–63.6) | | 73.0* (70.1–75.9) | | 67.7* (64.5–70.9) | | 60.3 (55.1–65.6) | |
| 2 RFs | 52.6 (48.8–56.3) | | 47.7 (42.4–52.9) | | 65.1* (58.6–71.5) | | 64.0* (58.7–69.2) | | 49.3 (39.7–59.0) | |
| 3 RFs | 55.6 (50.1–61.1) | | 52.8 (46.2–59.4) | | 72.5* (63.0–81.9) | | 66.7* (58.1–75.3) | | 52.9 (34.9–70.9) | |
| ≥4 RFs | 58.2 (52.3–64.0) | | 56.0 (48.5–63.5) | | 62.4 (51.5–73.4) | | 68.2 (57.8–78.6) | | 50.4 (26.8–73.9) | |
| **Pulmonary and cardiovascular** | **59.8 (57.5–62.2)** | | **56.5 (53.3–59.7)** | | **72.4* (68.6–76.2)** | | **67.3* (64.3–70.2)** | | **57.5 (51.8–63.2)** | |
| **Pulmonary** | **79.6 (77.1–82.2)** | | **77.3 (73.9–80.7)** | | **86.5* (83.2–89.9)** | | **85.4* (82.7–88.2)** | | **81.5 (74.8–88.3)** | |
| COPD^b^ | 54.8 (49.4–60.2) | | 52.8 (46.5–59.0) | | 62.7 (47.6–77.8) | | 70.7* (63.0–78.4) | | 48.6 (29.6–67.6) | |
| Asthma (current)^c^ | 84.3 (81.7–86.8) | | 82.8 (79.2–86.4) | | 88.3* (84.9–91.6) | | 87.1* (84.2–90.0) | | 82.4 (75.9–89.0) | |
| **Cardiovascular** | **34.0 (31.5–36.6)** | | **30.3 (27.1–33.4)** | | **50.1* (44.5–55.8)** | | **41.9* (37.4–46.4)** | | **29.8 (23.3–36.3)** | |
| CHF | 29.5 (24.3–34.8) | | 25.5 (18.8–32.1) | | 45.5* (35.2–55.9) | | 38.0* (30.5–45.5) | | 27.7 (14.4–41.0) | |
| CHD | 23.9 (19.5–28.2) | | 20.5 (15.2–25.9) | | 41.0* (32.1–50.0) | | 33.0* (25.6–40.5) | | 26.2 (15.3–37.1) | |
| Stroke | 32.2 (28.8–35.6) | | 28.9 (23.8–34.1) | | 45.9* (36.9–54.9) | | 39.1* (33.0–45.1) | | 26.4 (14.2–38.6) | |
| Angina pectoris | 42.2 (36.0–48.4) | | 39.4 (32.2–46.6) | | 59.1* (48.4–69.8) | | 50.4 (40.0–60.8) | | 35.8 (20.4–51.2) | |
| MI | 32.9 (29.1–36.8) | | 30.9 (25.6–36.1) | | 41.3* (32.9–49.7) | | 40.1 (31.7–48.4) | | 37.4 (24.2–50.7) | |
| **Endocrine and metabolic** | **50.4 (47.6–53.2)** | | **45.7 (41.8–49.7)** | | **63.1* (59.0–67.2)** | | **57.0* (53.8–60.2)** | | **51.5 (45.5–57.5)** | |
| Diabetes^d^ | 47.6 (45.0–50.3) | | 42.2 (38.2–46.1) | | 61.2* (56.8–65.7) | | 56.3* (52.9–59.7) | | 47.0 (40.4–53.6) | |
| Renal disease^e^ | N/A | | N/A | | N/A | | N/A | | N/A | |
| Liver disease  (current)^f^ | 61.2 (53.7–68.6) | | 60.0 (49.2–70.7) | | 66.4 (58.9–74.0) | | 54.4 (43.1–65.7) | | 64.0 (51.0–77.0) | |
| RFs, % (95% CI) | **By PIR** | | | | | | | | | |
|  | **≥5**  **(N=10,841,666)** | **4–<5**  **(N=4,840,077)** | | **3–<4**  **(N=6,418,387)** | | **2–<3**  **(N=8,818,228)** | | **1–<2**  **(N=12,846,534)** | | **<1**  **(N=9,548,521)** |
| **≥1 RF^a^** | **58.6 (54.3–62.9)** | **62.9 (56.4–69.5)** | | **58.3 (52.9–63.7)** | | **55.5 (51.2–59.8)** | | **56.7 (53.6–59.8)** | | **71.0* (66.3–75.7)** |
| 1 RF | 63.4 (58.8–67.9) | 66.9 (59.3–74.6) | | 62.6 (56.2–69.0) | | 58.4 (53.2–63.6) | | 60.0 (56.3–63.7) | | 72.4* (66.7–78.1) |
| 2 RFs | 46.7 (34.7–58.6) | 62.4* (51.2–73.7) | | 41.6 (32.0–51.2) | | 47.9 (38.3–57.4) | | 50.0 (42.9–57.2) | | 67.1* (59.8–74.3) |
| 3 RFs | 38.1 (19.9–56.3) | 45.0 (21.9–68.1) | | 61.0 (38.0–84.0) | | 51.5 (37.6–65.3) | | 51.5 (42.3–60.8) | | 76.4* (68.7–84.0) |
| ≥4 RFs | 54.9 (33.0–76.7) | 43.5 (16.3–70.7) | | 57.7 (37.3–78.1) | | 56.1 (37.2–75.1) | | 56.1 (47.4–64.9) | | 67.4 (58.0–76.7) |
| **Pulmonary and cardiovascular** | **56.7 (50.9–62.5)** | **64.7 (57.0–72.4)** | | **56.9 (50.4–63.4)** | | **56.2 (51.3–61.1)** | | **57.0 (53.5–60.5)** | | **71.7* (68.5–74.8)** |
| **Pulmonary** | **84.3 (79.4–89.3)** | **82.7 (75.0–90.4)** | | **75.2* (67.1–83.4)** | | **81.2 (75.7–86.6)** | | **76.1* (71.5–80.7)** | | **80.4 (75.9–84.8)** |
| COPD^b^ | 60.9 (39.4–82.4) | 44.9 (19.3–70.5) | | 38.8 (20.0–57.7) | | 51.8 (36.5–67.0) | | 55.3 (48.4–62.1) | | 67.0 (56.3–77.8) |
| Asthma (current)^c^ | 86.2 (81.2–91.2) | 86.4 (78.6–94.3) | | 81.7 (74.5–89.0) | | 86.5 (81.0–92.0) | | 81.9 (77.6–86.2) | | 83.5 (78.7–88.3) |
| **Cardiovascular** | **22.2 (15.4–29.0)** | **30.5 (19.5–41.5)** | | **34.0* (24.4–43.5)** | | **28.7 (23.9–33.4)** | | **34.0* (29.9–38.1)** | | **52.8* (48.2–57.4)** |
| CHF | 19.3 (1.8–36.9) | 29.7 (9.3–50.1) | | 30.4 (16.9–43.9) | | 18.4 (7.7–29.1) | | 29.8 (23.1–36.5) | | 42.5* (33.7–51.3) |
| CHD | 14.3 (5.7–22.8) | 28.6* (12.6–44.7) | | 17.0 (6.9–27.1) | | 18.5 (9.7–27.3) | | 22.4 (17.6–27.2) | | 43.6* (36.7–50.6) |
| Stroke | 19.9 (9.6–30.1) | 18.7 (4.2–33.3) | | 33.4 (17.7–49.1) | | 27.9 (17.7–38.0) | | 30.6 (23.9–37.2) | | 50.7* (42.9–58.6) |
| Angina pectoris | 30.0 (9.7–50.3) | 42.0 (21.4–62.6) | | 62.4* (41.5–83.4) | | 41.7 (29.6–53.8) | | 37.6 (27.4–47.7) | | 55.5* (45.4–65.6) |
| MI | 22.0 (9.5–34.6) | 25.0 (10.0–40.1) | | 35.0 (15.5–54.4) | | 27.1 (16.8–37.3) | | 27.3 (20.7–33.9) | | 58.3* (49.8–66.8) |
| **Endocrine and metabolic** | **50.6 (45.2–56.0)** | **46.3 (36.5–56.2)** | | **54.1 (46.9–61.2)** | | **43.3 (36.9–49.7)** | | **45.6 (41.0–50.2)** | | **62.0* (55.1–68.9)** |
| Diabetes^d^ | 47.0 (41.2–52.8) | 40.4 (30.7–50.1) | | 53.0 (45.8–60.3) | | 40.4 (32.9–47.8) | | 43.5 (38.7–48.3) | | 62.2* (57.4–67.0) |
| Renal disease^e^ | N/A | N/A | | N/A | | N/A | | N/A | | N/A |
| Liver disease  (current)^f^ | 66.4 (50.7–82.0) | 71.4 (53.3–89.4) | | 56.0 (36.8–75.1) | | 65.3 (49.6–81.0) | | 52.5 (40.4–64.6) | | 59.4 (38.8–80.1) |

Notes: Age at diagnosis for diagnosed estimates includes age at self-reported diagnosis among individuals who reported having a diagnosis of a RF in the interview component. Hispanic group defined in NHANES as Mexican American or other Hispanic. PIR calculated by NHANES by dividing family (or individual) income by the US Health and Human Services poverty guidelines relevant to the survey year; a ratio <1 represents family income below poverty level. Other race and ethnicity/multi-racial results and missing PIR results not presented.

^a^Summary measures of RFs include those measurable via NHANES: COPD, asthma (current), CHF, CHD, stroke, angina pectoris, MI, diabetes, and/or liver disease (current). Age at self-reported diagnosis is not available for renal disease.

^b^Defined as a “yes” response to any of the following questionnaire items: MCQ160o (Has a doctor or other health professional ever told [you/SP] that [you/s/he] had COPD? 2013–2014 and 2015–2016 only) or MCQ170k (Do you still have chronic bronchitis? 2011–2012, 2013–2014 and 2015–2016 only), MCQ160g (Has a doctor or other health professional ever told [you/SP] that [you/s/he] had emphysema? 2011–2012, 2013–2014 and 2015–2016 only), or MCQ160p ([Have you/Has SP] ever been told by a doctor or other health professional that [you/he/she] had chronic obstructive pulmonary disease or COPD, emphysema, or chronic bronchitis? 2017–March 2020 only).

^c^Only asked among respondents who answered “yes” to having ever received a diagnosis of asthma. Respondents who did not report ever receiving a diagnosis of asthma are included in the “no” category.

^d^Respondents who reported having borderline diabetes are included in the “no” category.

^e^Age at self-reported diagnosis is not available for renal disease.

^f^Only asked among respondents who answered “yes” to having ever received a diagnosis of liver disease. Respondents who did not report ever receiving a diagnosis of liver disease are included in the “no” category.

*Statistically significant (p<0.05) based on pairwise chi-square analysis on 2x2 tables comparing proportion of respondents with each RF in respective group to the reference group (i.e., non-Hispanic White or PIR ≥5).

Abbreviations: CHD, coronary heart disease; CHF, congestive heart failure; CI, confidence interval; COPD, chronic obstructive pulmonary disease; MI, myocardial infarction; NHANES, National Health and Nutrition Examination Survey; PIR, poverty income ratio; RF, risk factor; RSV, respiratory syncytial virus; US, United States.
